# Genome-wide identification of the expansin gene family reveals that *expansin* genes are involved in fibre cell growth in cotton

**DOI:** 10.1186/s12870-020-02362-y

**Published:** 2020-05-19

**Authors:** Li-Min Lv, Dong-Yun Zuo, Xing-Fen Wang, Hai-Liang Cheng, You-Ping Zhang, Qiao-Lian Wang, Guo-Li Song, Zhi-Ying Ma

**Affiliations:** 1 Hebei Research Base, State Key Laboratory of Cotton Biology in China, Hebei Agricultural University, Baoding, 071001 China; 2 State Key Laboratory of Cotton Biology, Institute of Cotton Research, Chinese Academy of Agricultural Sciences (CAAS), Anyang, 455000 China; 3 North China Key Laboratory for Crop Germplasm Resources of the Education Ministry, Hebei Agricultural University, Baoding, 071001 China; 4 Zhengzhou Research Base, State Key Laboratory of Cotton Biology, Zhengzhou University, Zhengzhou, China

**Keywords:** Expansins, Gene family, Fibre, Gene expression profiles, *Gossypium hirsutum*

## Abstract

**Background:**

Expansins (*EXPs*), a group of proteins that loosen plant cell walls and cellulosic materials, are involved in regulating cell growth and diverse developmental processes in plants. However, the biological functions of this gene family in cotton are still unknown.

**Results:**

In this paper, we identified a total of 93 *expansin* genes in *Gossypium hirsutum*. These genes were classified into four subfamilies, including 67 *GhEXPAs*, 8 *GhEXPBs*, 6 *GhEXLAs*, and 12 *GhEXLBs*, and divided into 15 subgroups. The 93 *expansin* genes are distributed over 24 chromosomes, excluding Ghir_A02 and Ghir_D06. All *GhEXP* genes contain multiple exons, and each GhEXP protein has multiple conserved motifs. Transcript profiling and qPCR analysis revealed that the *expansin* genes have distinct expression patterns among different stages of cotton fibre development. Among them, 3 genes (*GhEXPA4o*, *GhEXPA1A*, and *GhEXPA8h*) were highly expressed in the initiation stage, 9 genes (*GhEXPA4a*, *GhEXPA13a*, *GhEXPA4f*, *GhEXPA4q*, *GhEXPA8f*, *GhEXPA2*, *GhEXPA8g*, *GhEXPA8a*, and *GhEXPA4n*) had high expression during the fast elongation stage, and *GhEXLA1c* and *GhEXLA1f* were preferentially expressed in the transition stage of fibre development.

**Conclusions:**

Our results provide a solid basis for further elucidation of the biological functions of *expansin* genes in relation to cotton fibre development and valuable genetic resources for future crop improvement.

## Background

Expansins are a kind of cell wall-loosening protein that are widely present in higher plants, bacteria and fungi. Expansins may unlock the network of wall polysaccharides without lytic activity, permitting turgor-driven cell enlargement [1]. Plant expansins are usually 250 to 275 amino acid residues in length, and the majority have a signal peptide in the N terminus; the signal peptides are usually 20 to 30 amino acid residues long [2, 3]. Typical structures of plant expansins are torpedo-shaped proteins containing two domains, domain I and domain II. Domain I is a six-stranded double-psi beta-barrel (DPBB), which has similar characteristics to the catalytic domain of glycoside hydrolase family 45 (GH45) proteins and contains a conserved His-Phe-Asp (HFD). The DPBB domain does not, however, possess the same catalytic activity as GH45. Domain II is homologous to group-2 grass pollen allergens [2], and it was recently classified as a family-63 carbohydrate binding module (CBM63) [2, 4].

The plant expansin superfamily is divided into four subfamilies, which include α-expansin (EXPA), β-expansin (EXPB), expansin-like A (EXLA), and expansin-like B (EXLB). Expansins were first identified as endogenous proteins inducing cell wall extension in plants by the McQueen-Mason group [5]. It has been shown that they can participate in many developmental processes and function in cell growth and enlargement, pollen tube invasion of the stigma (in grasses), wall disassembly during fruit ripening, abscission, stress resistance, and other cell separation events [1, 2, 6, 7].

Cotton fibres are single-celled trichomes that differentiate from the ovule epidermis, which is a powerful cell expansion and wall biogenesis research model system [8]. The process of cotton fibre development can be divided into five stages: initiation, elongation, transition, secondary wall synthesis and maturation [9]. Some *expansin* genes preferentially expressed in cotton fibres were isolated and identified using several different approaches, including cDNA arrays, subtractive PCR, RT-PCR, and so on [10, 11]. The functions of *expansin* genes in cotton fibre development have been further investigated. Over-expression of *GhEXPA8* can improve cotton fibre length and micronaire value [12]. *GbEXPATR*, a *Gossypium barbadense*-specific expansin, can also enhance cotton fibre elongation through cell wall restructuring [13]. *GhRDL1* is localized in the cell wall and interacts with *GhEXPA1*, and cotton plants overexpressing *GhRDL1* and *GhEXPA1* have an increased fibre length and produce many more cotton bolls [14]. *GhEXPA1* expression levels are regulated by the transcription factor *GhHOX3*, which can promote cotton fibre elongation [15].

Thus, expansins play an important role in cotton fibre development. The cotton genome has been sequenced and re-sequenced in succession [16–19]. These genomic data make genome-wide identification of gene families possible. *Gossypium hirsutum* possesses a complex allotetraploid genome (AADD; 2n = 52), which resulted from the doubling of two diploid cotton genomes, specifically, those of *Gossypium. arboreum* (AA; 2n = 26) and *Gossypium raimondii* (DD; 2n = 26) [17, 20, 21]. At present, upland cotton accounts for 90% of natural fibre production worldwide. Hence, we mainly examined the whole *expansin* gene family of *G. hirsutum* in this paper. This research can provide genome-wide information on cotton *expansin* genes and promote further investigations of the biological function of *expansin* genes during cotton fibre development and other developmental processes.

## Results

### Identification and sequence analysis of the cotton expansin gene family

We have identified the expansin gene family in the *G. hirsutum* genome. As a result, 98 candidate *expansin* genes were initially obtained. According to the analysis of conserved expansin domains, 93 *expansin* genes with both DPBB-1 (domain I) and Pollen_allerg_1 (domain II) domains were ultimately identified for further analysis. Each *expansin* gene was named according to nomenclature guidelines. The detailed results are shown in Table S1. The expansin gene family contained four subfamilies, including EXPA, EXPB, EXLA, and EXLB. For the expansin gene family in *G. arboreum* and *G. raimondii*. The same analysis methods were performed. As a result, 49 and 45 *expansin* genes were identified in the *G. arboreum* and *G. raimondii* genomes, respectively. These *expansin* genes were also divided into 4 subfamilies. The detailed results are shown in Table S2 and Table S3.

We analysed the biochemical properties of expansin proteins (Table S1). The pI values of expansin family members ranged from 4.65 (GhEXLB1l) to 12.01 (GhEXPA4c), with an average of 8.47. The pI values of all EXPA and EXLA members were above 7.0, except for those of GhEXPA8b and GhEXPA7d. However, the pI values of all EXPBs and EXLBs were below 7.0, except for those of GhEXPB3a, GhEXPB3b, GhEXPB1a, GhEXPB1b, GhEXLB1d, and GhEXLB1j (Additional file 1: Table S1). The average MW of expansin family members was 27.42 kD, ranging from 14.29 (GhEXPA17e) to 41.53 (GhEXPA5e) kD. The length of expansin protein sequences ranged from 150 (GhEXLA17e) amino acids (aa) to 366 aa (GhEXPA5e), and the signal peptide length ranged from 17 (GhEXPA15d, GhEXPA15g and GhEXLA1d) to 35 (GhEXLA1a) aa (Additional file 1: Table S1).

The multiple sequence alignment results of 93 expansin proteins from *G. hirsutum* showed that they had similar sequence characteristics: the majority of them consisted of a signal peptide and conserved domains I and II (Additional file 2: Figure S1), which was consistent with the findings of a previous study [3]. The amino acid sequence of domain I was more conserved than that of domain II, especially among EXPA members (Additional file 2: Figure S1). Notably, almost all of the EXPAs (excluding GhEXPA13a, GhEXPA13b, and GhEXPA15d) and three EXLA members (GhEXLA17a, GhEXLA17b, and GhEXLA17c) contained a conserved motif (HFD) in domain I (Additional file 2: Figure S1). Members of EXPB, EXLB, and the six other EXLA members did not have the HFD motif. Six EXLAs contained an extra segment named the EXLA extension of the C terminus. The EXLA extension sequence feature was found only in the EXLA subfamily, and the amino acid sequences of the EXLA extension were as follows: “DIAK(Q)EGCS(F)P(H)CDD(Y)S(G)H(N)WR(−)”. In addition, a conserved motif named BOX 1 was found in almost all the expansin members (Additional file 2: Figure S1).

### Phylogenetic relationships, gene structure and protein motifs of cotton *expansin* genes

**Fig. 1:**
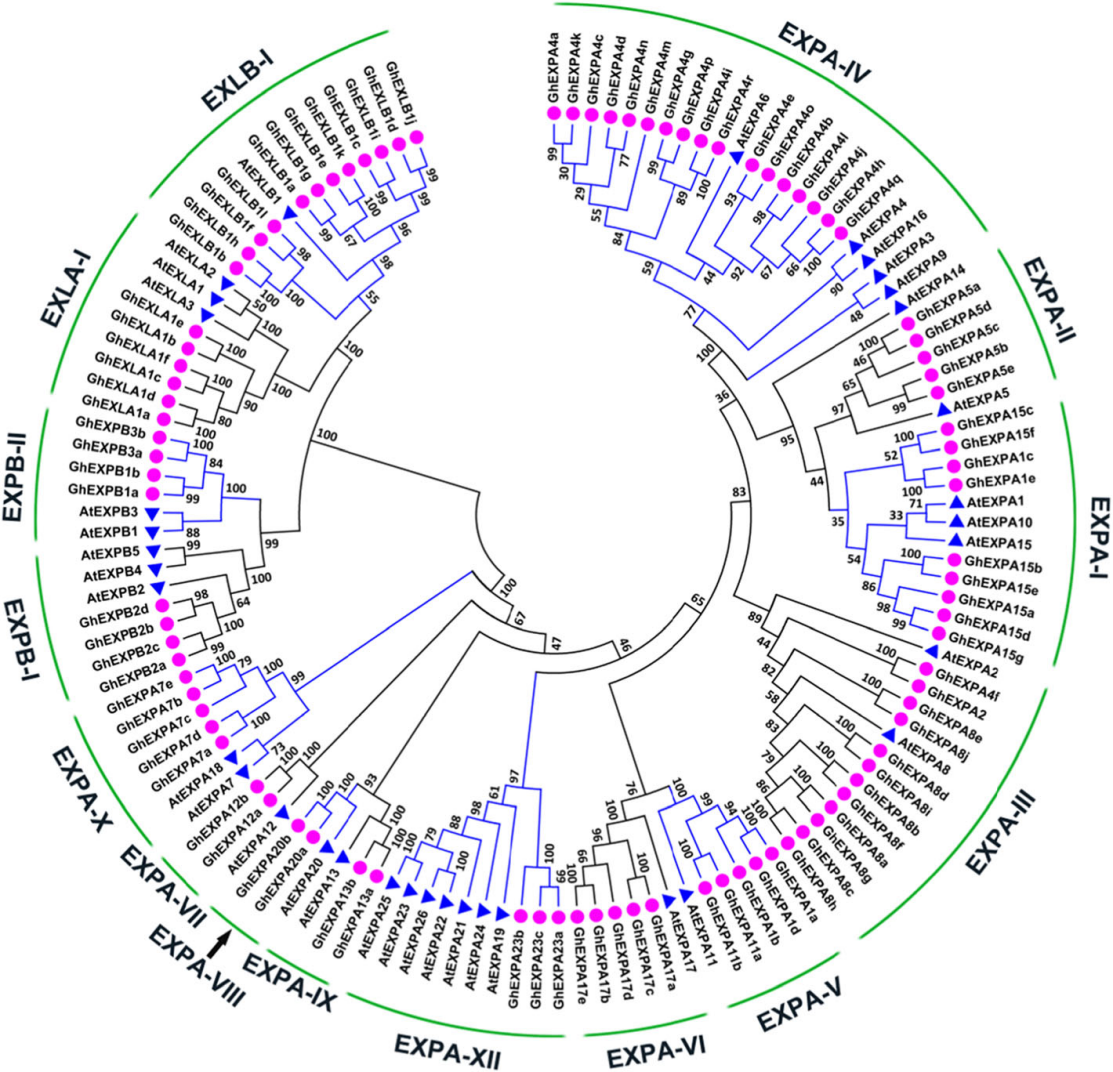
Phylogenetic analysis and subfamily classification of the *expansin* genes in cotton (*GhEXPs*). The phylogenetic tree was constructed with MEGA 6.0 software using the neighbour-joining (NJ) method with 1000 bootstrap replicates. The pink solid circles represent the cotton *expansin* genes from *Gossypium hirsutum*; the blue solid triangles represent the *expansin* genes from *Arabidopsis thaliana*. Gh, *Gossypium hirsutum;* At, *Arabidopsis thaliana*

To evaluate the evolutionary relationships of cotton expansins, a phylogenetic tree was constructed. The expansins were divided into four major subfamilies, namely, EXPA, EXPB, EXLA and EXLB. The EXPA subfamily was the largest group, with 67 members, and the other subfamilies contained eight (EXPB), six (EXLA), or 12 (EXLB) members. The four expansin subfamilies comprised 15 subgroups (Fig. 1). We discovered that EXPA-IV was the largest subgroup, which included 17 expansin members, and EXPA-VII, EXPA-VIII, and EXPA-IX were the smallest subgroups, with only two expansin members each.

**Fig. 2:**
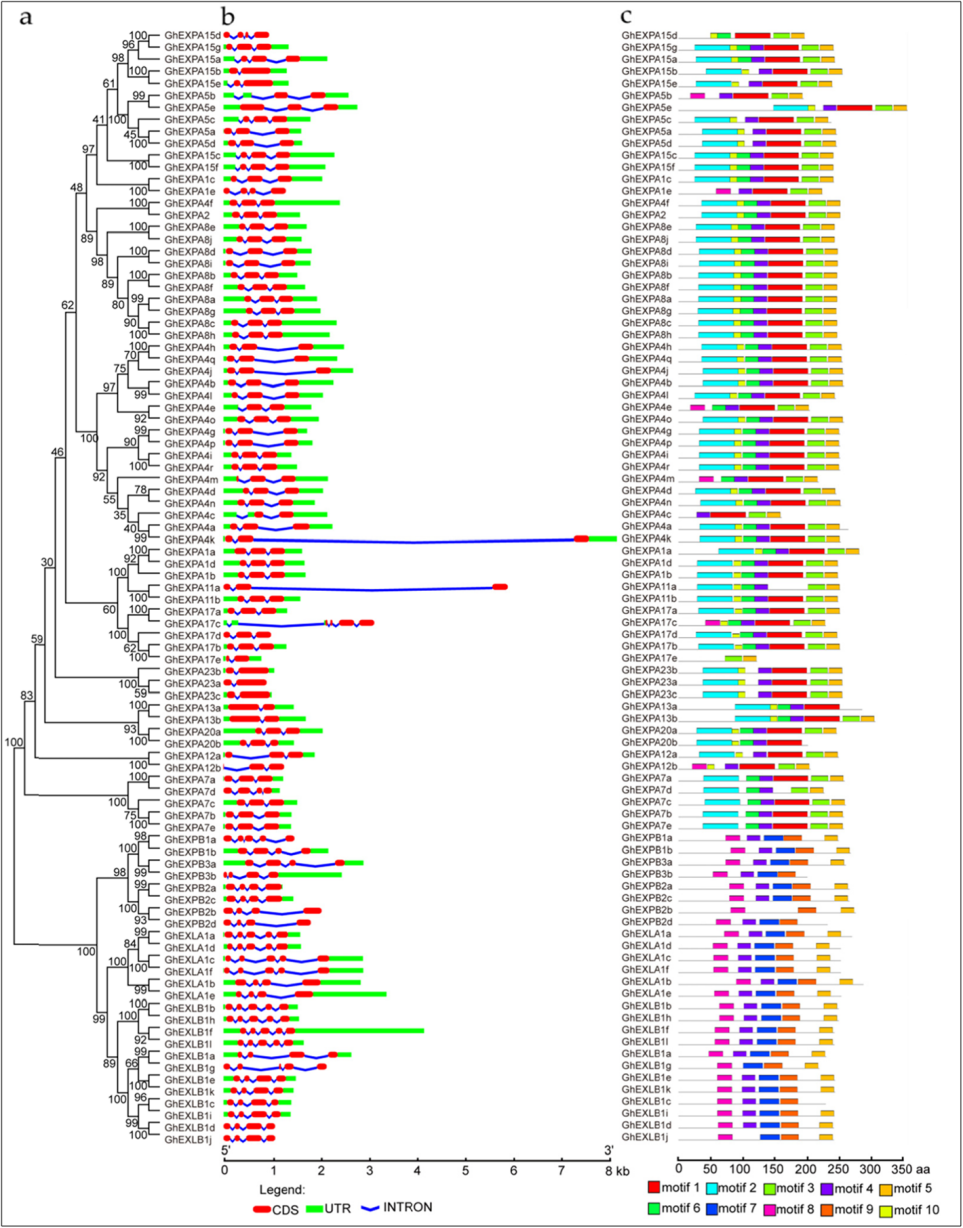
Phylogenetic relationships, gene structure, and protein domain architecture of *GhEXP* genes. **a** Phylogenetic relationships of 93 GhEXP proteins. The phylogenetic tree was constructed with MEGA 6.0 software using the neighbour-joining (NJ) method with 1000 bootstrap replicates. **b** Gene structure (exon-intron organization) analysis of *GhEXPs*. The gene structures were drawn online with Gene Structure Display Server 2.0 [22]. The CDSs, introns, and UTRs are marked with red boxes, blue lines, and green boxes, respectively. The scale bar is shown at the bottom. **c** Analysis of conserved domains of the GhEXP proteins. Differently coloured boxes represent different conserved motifs of GhEXP proteins

The results of gene structure (exon-intron organization) analysis showed that the expansin members included two to five exons, and the same subfamilies had similar characteristics of exon types (Fig. 2a, b). Most of the *EXPA* members had three exons (51 of 67 *EXPA* members). Twelve *EXPAs* had two exons, and four *EXPAs* had four exons. All members of the *EXPB* subfamily had four exons except for *GhEXPB1a* (five exons). Four *EXLA* members contained five exons, and two members had four exons. *EXLB* members had four (seven *EXLBs*) or five exons (five *EXLBs*).

We identified the conserved motifs in expansin proteins. As a result, a total of ten distinct motifs were identified (Fig. 2c, Additional file 2: Figure S2). The motifs of all cotton expansins had unifying features; for example, each expansin protein contained motif 5, and all of them contained motif 4, except for GhEXPA15d, GhEXPA17e, and GhEXLB1j. In addition, the type, arrangement, and number of motifs were similar within the same subfamily. More than half of the EXPA members (38/67) had seven motifs, and 21 members had six motifs. The EXPB, EXLA, and EXLB subfamilies possessed similar motif characteristics, and most of them contained five motifs (motifs 4, 5, 7, 8, and 9). GhEXPB2d and GhEXPB3b of the EXPB subfamily included four motifs, and three members (GhEXLB1g, GhEXLB1c, and GhEXLB1j) of EXLB also had the same number of motifs. These results showed that the EXPB, EXLA, and EXLB subfamilies had close evolutionary relationships. The similarities between gene structures and sequence motifs implied that cotton expansin family genes underwent duplication over evolutionary time.

### Chromosomal location and collinearity analysis of the expansin gene family

**Fig. 3:**
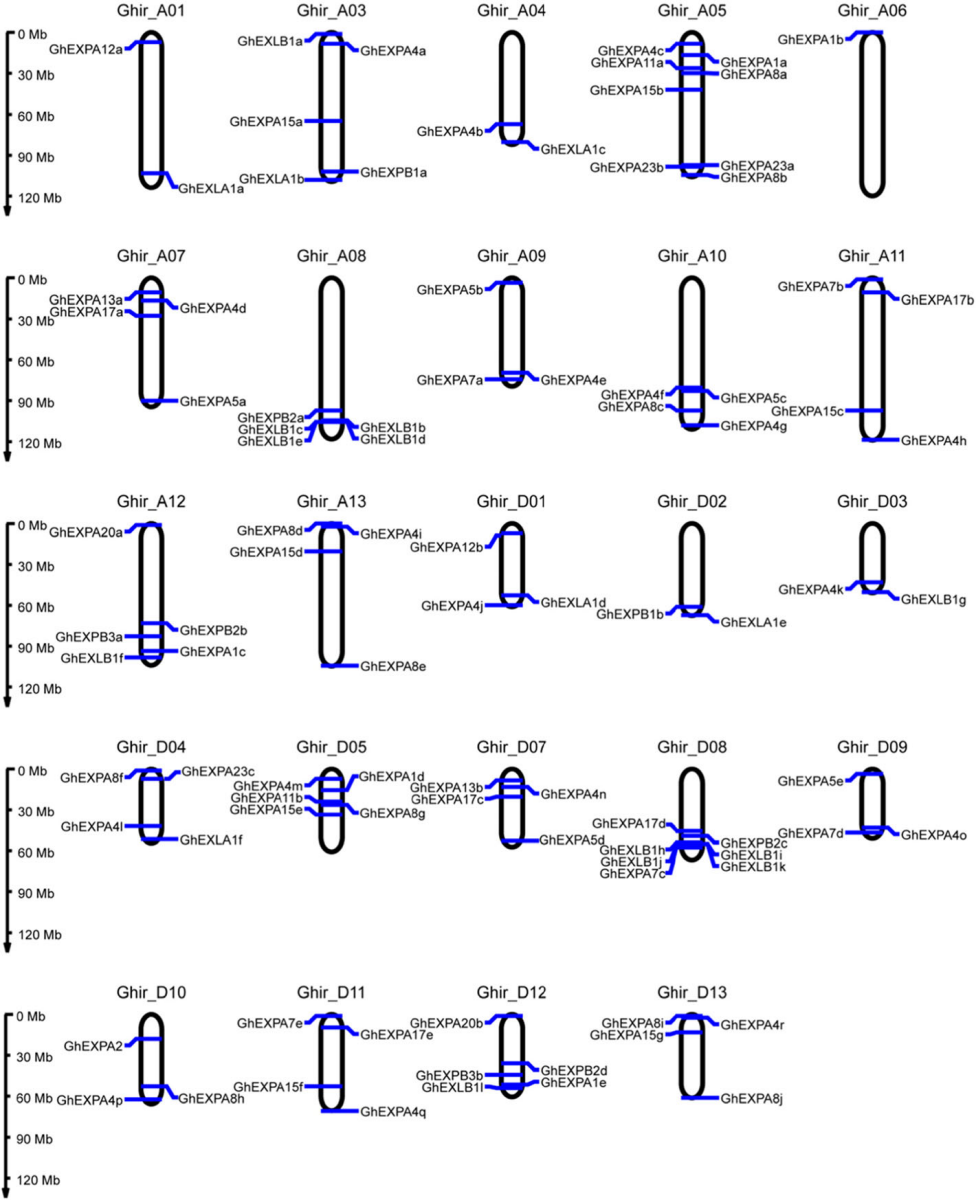
Chromosomal distribution of *GhEXP* genes. The chromosome name is above each chromosome, and the blue lines on the chromosome are the gene names

**Fig. 4:**
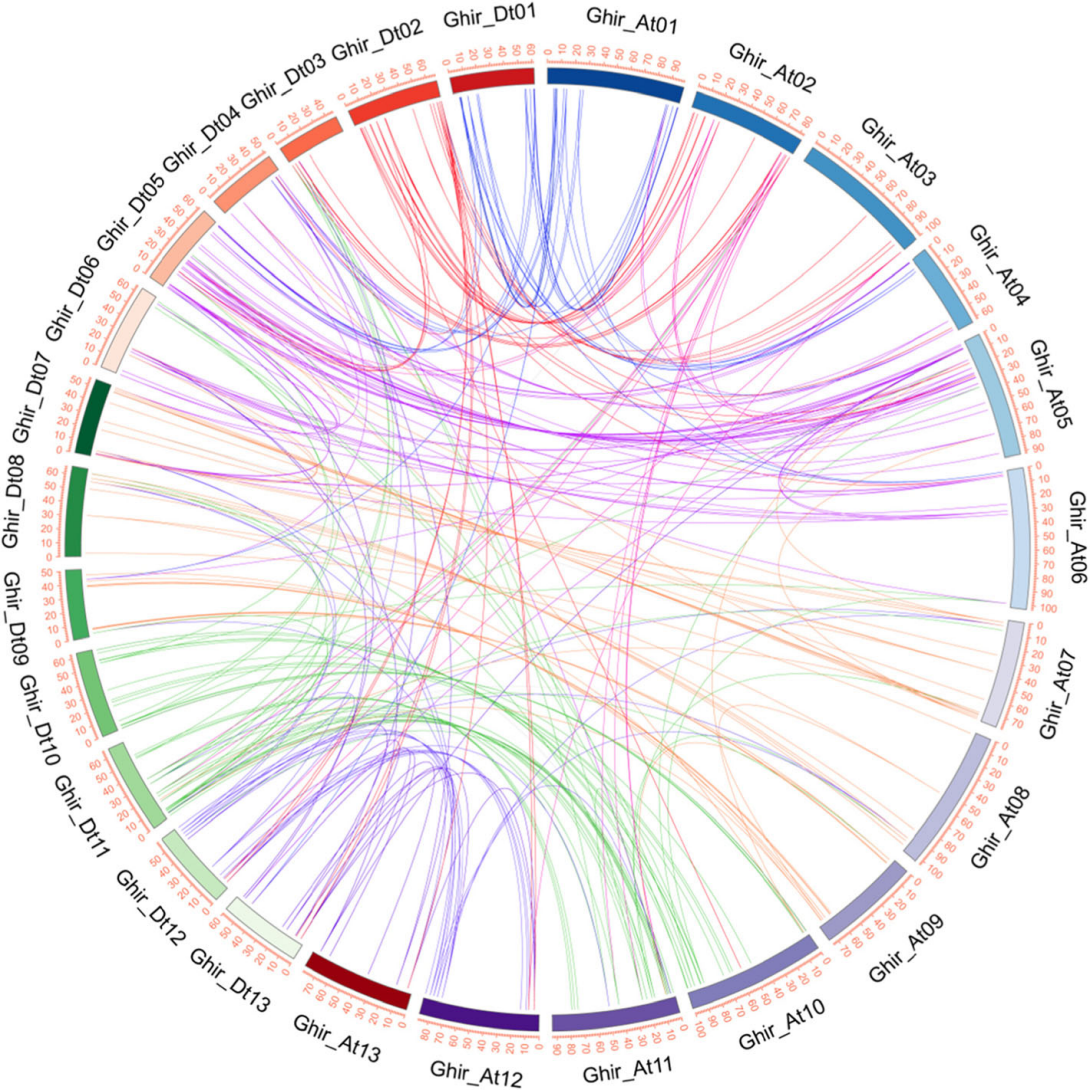
The collinearity relationships of *GhEXP* genes in upland cotton. The inner coloured lines show syntenic blocks in homoeologous chromosomes among cotton *expansin* genes and between the A and D sub-genomes

The chromosomal location of *GhEXP* genes was identified in *G. hirsutum*. The results are shown in Fig. 3. A total of 93 *expansin* genes were distributed on 24 chromosomes, excluding Ghir_A02 and Ghir_D06. The chromosome Ghir_A05 contained eight *expansin* genes, whereas Ghir_A06 included only one *expansin* gene. The numbers of *expansin* genes located on other chromosomes ranged from two to seven. In addition, some of the *expansin* genes were located on the chromosome in clusters; for example, both Ghir_A08 and Ghir_D08 possessed a gene cluster with four distinct EXLBs (Fig. 3). These results showed that the *expansin* genes were unevenly distributed on each chromosome. Collinearity analysis showed that *expansin* genes were frequently collinear between the A and D sub-genomes (Fig. 4), which indicated that *expansin* genes with collinear relationships may have similar functions.

### Investigation of *cis*-acting elements in the promoter regions of *expansin* genes

We identified the *cis*-acting regulatory elements of the cotton expansin gene family. The results showed that the *cis*-acting regulatory elements of *expansin* genes were extremely diverse (Additional file 1: Table S4; Table S5). These elements were divided into 7 categories and 111 types, including 31 light-responsive elements, 7 development-related elements, 13 hormone-responsive elements, 5 environmental stress-related elements, 3 promoter-related elements, 7 site-binding-related elements and 44 other elements (no functions). Among them, the light and hormone responsive types were especially abundant (Additional file 1: Table S4; Table S5).

All 93 *GhEXP* genes possessed 15,200 elements, including 1268 light-responsive elements, 144 development-related elements, 779 hormone-responsive elements, 409 environmental stress-related elements, 9416 promoter-related elements, 81 site-binding-related elements and 3103 other elements (Additional file 1: Table S4). Out of 93 *GhEXP* genes, 83 possessed a Box 4 element (part of a conserved DNA module involved in light responsiveness), 70 possessed a GT1 motif (light-responsive element), 57 had a G-box (*cis*-acting regulatory element involved in light responsiveness) with 70 enriched ABRE elements (the *cis*-acting element involved in abscisic acid responsiveness), 75 contained an ERE, 56 had a TGACG motif as well as a TGACG motif, which are the *cis*-acting regulatory elements involved in MeJA responsiveness, 73 harboured an ARE (cis-acting regulatory element essential for anaerobic induction), and 40 possessed an MBS (MYB binding site involved in drought inducibility). Moreover, these relatively abundant elements were also more conserved among the *GhEXP* gene family. In addition, all the *GhEXP* genes contained a CAAT-box and TATA-box, which are the core elements of the promoter in eukaryotes, and these genes contained the largest numbers of these elements (Additional file 1: Table S5).

### Expression patterns of the *expansin* genes in cotton fibre

**Fig. 5:**
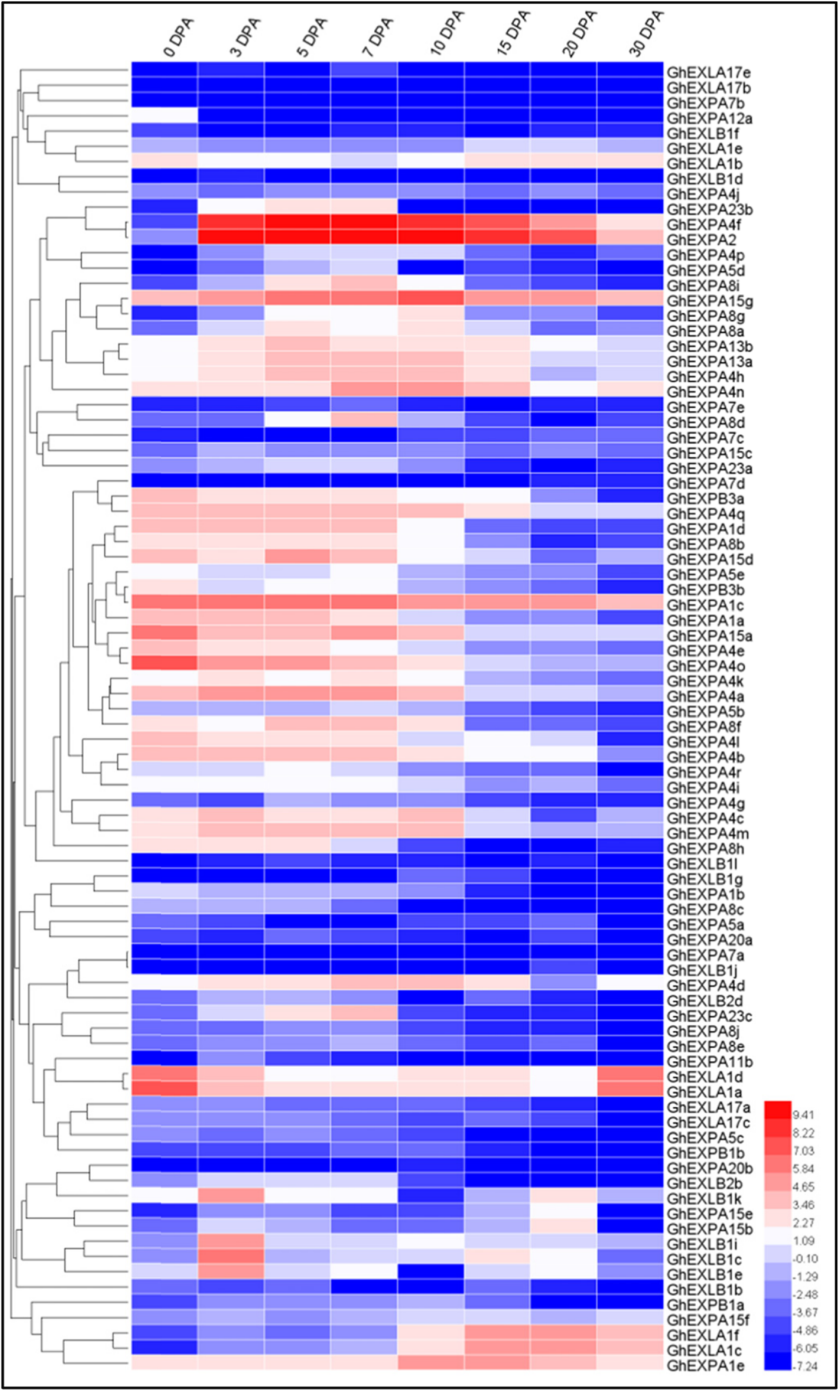
Expression profiles of *GhEXP* genes at different cotton fibre developmental stages. The heat map was constructed based on RNA-seq data. Different colours represent the different expression levels of *GhEXP* genes. The legend represents the logarithm-transformed values of log2. DPA, day post anthesis; FPKM, fragments per kilobase of transcript per million mapped reads

To comprehensively investigate the temporal expression patterns of the cotton expansin gene family, fibre samples at different developmental stages were used for transcriptome analysis. A heat map was constructed with these transcriptome data (Fig. 5). The 86 *expansin* genes displayed different expression patterns. The remaining seven *expansin* genes were not detected in the transcriptome data. Although the expression patterns of *expansin* genes displayed obvious differences, clustered *expansin* genes generally possessed similar expression patterns. For example, *GhEXPA1d*, *GhEXPA15d*, *GhEXPA15a*, *GhEXPA4o*, *GhEXPA4a* and *GhEXPA4b* were the preferentially expressed genes during the fibre initiation and elongation stages (0 to 15 DPA), whereas *GhEXLA1f* and *GhEXLA1c* had higher expression during the middle and later cotton fibre developmental stages (after 15 DPA). In addition, *GhEXPA4f* and *GhEXPA2*, two homologous genes located on the A and D sub-genomes, respectively, were sharply up-regulated from 3 DPA, with very similar expression patterns (Fig. 5), suggesting that they may have similar or complementary functions in cotton fibre development. To verify our transcriptome results, the *GhEXP* gene expression profiles were further confirmed using publicly available RNA-seq data. The expression profiles of *GhEXP* genes were generally consistent with our transcriptome results (Fig. 5 and Figure S3).

To avoid missing possible important *expansin* genes, we also analysed the transcript levels of seven *expansin* genes that were not detected in the transcriptome data (Fig. 5; Table S1). qRT-PCR showed that the seven *expansin* genes were scarcely expressed, except for *GhEXLB1h*, with low expression levels in ovules and fibres. In addition, we found that these genes can be detected in other tissues, but their expression levels were not high (Additional file 2: Figure S4).

### qRT-PCR analysis of the special *expansin* genes in cotton fibres

**Fig. 6:**
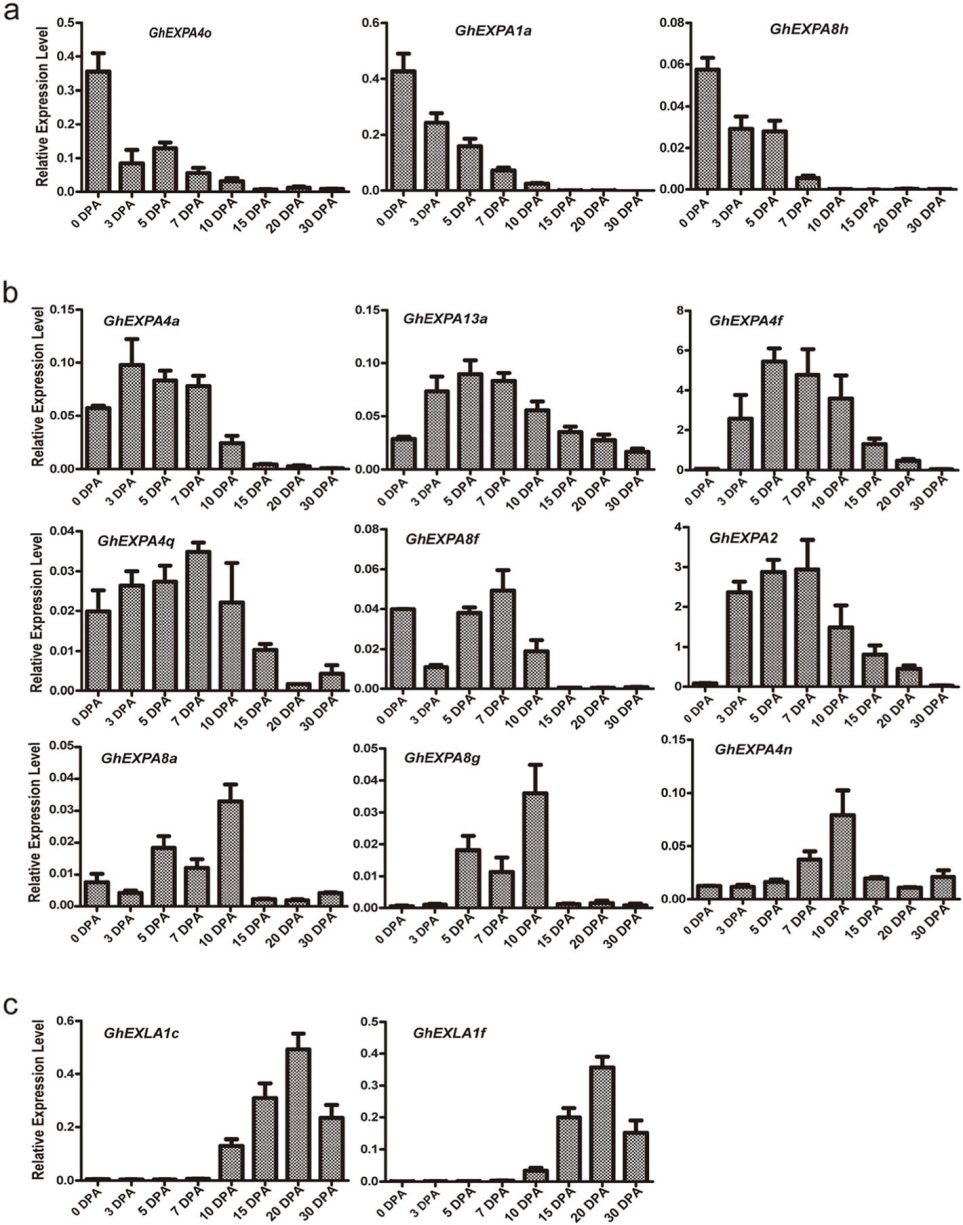
The expression patterns of 14 *GhEXPs* at different developmental stages of cotton fibres. **a** Expression profiles of three *GhEXP* genes highly expressed in the fibre initiation period. **b** Expression profiles of nine *GhEXP* genes highly expressed in the fibre elongation stage. **c** Expression profiles of two *GhEXP* genes highly expressed at the secondary wall synthesis stage. qRT-PCR experiments were performed with three independent replicates, and the error bars in this figure represent the SDs from three independent experiments

To further identify the key *expansin* genes involved in fibre cell growth, 14 *expansin* genes that are predominantly expressed in different developmental stages of cotton fibres were selected to verify their expression level using a qRT-PCR experiment. These *expansin* genes were evidently up-regulated at the initiation, elongation, or transition stage (Fig. 6) and displayed almost consistent expression tendencies when compared to those in the transcriptome data (Additional file 2: Figure S5).

We found that *GhEXPA4o*, *GhEXPA1a*, and *GhEXPA8h* were predominantly expressed at 0 DPA (Fig. 6a), suggesting that these three genes may function in the initial developmental stage of fibre cells. Nine *expansin* genes showed higher expression levels at the fibre elongation stage, with distinct expression characteristics (Fig. 6b). The expression of *GhEXPA4a* reached a peak at 3 DPA, and that of *GhEXPA13a* and *GhEXPA4f* peaked at 5 DPA. The expression levels of *GhEXPA4q*, *GhEXPA8f*, and *GhEXPA2* were the highest at 7 DPA, and those of *GhEXPA8g*, *GhEXPA8a*, and *GhEXPA4n* peaked at 10 DPA (Fig. 6b). *GhEXPA4f* and *GhEXPA2* are homologous genes in allotetraploid cotton species that are located in the A and D sub-genomes of the 10th chromosome, respectively, and both genes have specific expression in cotton fibre cells. Moreover, we found that *GhEXPA8a* and *GhEXPA8g* were important genes during cotton fibre elongation. These results revealed that the expression peaks of the majority of genes appeared from 7 to 10 DPA, which are usually called the fast elongation stages. In addition, we obtained two *expansin* genes that were predominantly expressed at transition stages, named *GhEXLA1c* and *GhEXLA1f* (Fig. 6c). Both of them belong to the EXLA subfamily and have unclear biological roles. The expression levels of *GhEXLA1c* and *GhEXLA1f* were the highest at 20 DPA, which is the transition stage of fibre cells from fast elongation to secondary cell wall synthesis.

To better understand the potential functions of 14 *expansin* genes, their expression profiles were detected in 11 different tissues, including roots, hypocotyls, stems, leaves, calycles, petals, pollen, stigmas, and fibres at 0 DPA, 10 DPA and 20 DPA. The results showed that these genes presented distinct but partially overlapping expression patterns (Additional file 2: Figure S6).

## Discussion

In this paper, we first reported on the expansin gene family in upland cotton, which included 93 members. All of the genes had two conserved domains, DPBB_1 and Pollen_allerg_1, consistent with results in other plants, such as *A. thaliana* [3], tobacco [22], tomato [23] and Chinese jujube [24]. Therefore, they are typical plant expansin proteins [2]. Phylogenetic analysis revealed that the 93 cotton expansins were divided into 15 subgroups of four subfamilies (Fig. 1). The number of expansin subgroups was consistent with the number of expansin ancestors including 15 to 17 *expansin* genes, and each of these ancestors evolved into an extant clade in the phylogenetic tree [25]. Thus, we speculated that each clade of the existing cotton expansin family might be extended by each clade ancestor. In addition, cotton *expansin* genes within every subfamily had structural similarity, and they also showed structural differences among the four subfamilies (Fig. 2b). The structural and evolutionary ancestor characteristics were consistent with those of other plant expansin gene families [22–24]. In the same subfamily category and even subgroup, most members had almost the same conserved gene structure and motif distribution (Fig. 2 b, c), further confirming their close evolutionary relationships and phylogenetic classification [26].

Our study showed that the *EXPA* subfamily genes in cotton were significantly expanded, including 67 total *EXPAs* (Fig. 1); this expansion in *EXPAs* suggests important functions of this kind of *expansin* gene in cotton growth and development. Conversely, there were fewer members of the other three expansin subfamilies relative to *EXPAs*: 8 *EXPBs*, 6 *EXLAs* and 12 *EXLBs*. The proportion of cotton *expansin* genes in each subfamily was almost consistent with that in other eudicots, such as *A. thaliana* (26 *EXPAs*, 6 *EXPBs*, 3 *EXLAs*, and 1 *EXLB*), grape (20 *EXPAs*, 4 *EXPBs*, 1 *EXLA*, and 4 *EXLBs*), jujube (19 *EXPAs*, 3 *EXPBs*, 1 *EXLA* and 7 *EXLBs*), and Chinese cabbage (39 *EXPAs*, 9 *EXPBs*, 2 *EXLAs*, and 3 *EXLBs*) [2, 3, 24, 27, 28]. The proportions are different in monocotyledons, such as rice (33 *EXPAs*, 18 *EXPBs*, 4 *EXLAs*, and 1 *EXLB*) and maize (36 *EXPAs*, 48 *EXPBs* and 4 *EXLAs*) [3, 29], with the most significant difference being that the *EXPBs* are more numerous in monocotyledons than in eudicots [2]. Furthermore, the number of *GhEXP* genes (93) was generally consistent with the sum of 49 *GaEXPs* and 45 *GrEXPs*. By comparative analysis, we found that 4 expansin subfamilies also existed in *G. arboreum* (38 *EXPAs*, 4 *EXPBs*, 2 *EXLAs*, and 5 *EXLBs*) and *G. raimondii* (33 *EXPAs*, 4 *EXPBs*, 3 *EXLAs*, and 5 *EXLBs*) (Additional file 1: Table S2; Additional file 1: Table S3). The results indicated that the A and D genomes of the two ancestral species were the donors of the modern allotetraploid genome of *G. hirsutum* [20]. These results provide significant insights into the evolution and functions of *expansin* genes in cotton.

Based on the expression profiles of *expansin* genes, we obtained 14 predominantly expressed genes in distinct stages of cotton fibre development, including 12 *EXPAs* and 2 *EXLAs* (Fig. 6), excluding *EXPBs* and *EXLBs*. Three *EXPA* genes, *GhEXPA4o*, *GhEXPA1a*, and *GhEXPA8h*, were first obtained in the early phase of fibre development and displayed high expression levels in qRT-PCR (Fig. 6a); however, their role in the initial stages of fibre development still needs to be clarified. Moreover, we also obtained nine *expansin* genes with higher expression levels in the elongation stages (Fig. 6b). Among the nine *expansin* genes, *GhEXPA4f* and *GhEXPA2* had the highest transcriptional levels during cotton fibre development, and they were the most preferentially expressed genes in different tissues (Fig. 6b; Additional file 2: Figure S6b). The expression level of *GhEXPA4f* was highly consistent with that reported for *GhExp1*, which was high in the fibre [30]. Its homologous gene *GhEXPA2* showed a similar expression pattern, and this result was nearly identical to that for the *GhExp2* expression level [30]. Transgenic plants with *GhEXPA1* and its partner *GhRDL1* exhibit improved cotton fibre yield [14], and overexpression of *GhEXPA8* can improve cotton fibre length [12]. By comparative analysis of gene sequences, we confirmed that *GhEXPA4f* (name in this study, GhirA10G15240), *GhExp1* [30] and *GhEXPA1* [14] are the same genes, as well as *GhEXPA2* (name in this study, GhirD10G12330) and *GhEXPA8*. Our qRT-PCR results also revealed the importance of the two *expansin* genes in cotton development. For the other seven new *expansin* genes, which were predominantly expressed in the elongation stages of cotton fibre development, the functions of these genes in terms of promoting fibre elongation need to be further studied. Interestingly, we found that *GhEXPA8a* and *GhEXP8g* are homologous with *AtEXP8* in *A. thaliana* and that *AtEXP8* can promote hypocotyl elongation in *A. thaliana* [31]. This result implies that *GhEXPA8a* and *GhEXP8g* can promote fibre cell elongation in cotton. The functional mechanism of *GhEXPA8a* and *GhEXP8g* will be one of our important research topics in the future. The above-mentioned *expansin* genes were the members of the *EXPA* subfamily that were expressed during the initial and elongation stages of fibre development. This may be because there are more members of the *EXPA* (67/93) subfamily in the expansin family. Moreover, these data also suggested that *expansin* genes of the EXPA subfamily are essential in cotton fibre development.

EXLA and EXLB were two smaller expansin subfamilies. Phylogenetic analysis showed that these proteins constitute separate and well-resolved groups; however, their biological functions are uncertain [2]. In this paper, we found two *EXLA* genes, referred to as *GhEXLA1c* and *GhEXLA1f*, with high expression at 20 DPA (Fig. 6c), which is the transition stage of fibre cells from fast elongation to secondary cell wall synthesis. These results suggested that they were important genes during the transition stage, in which cellulose synthesis is performed in preparation for the secondary wall thickening period. At present, there are relatively few studies on EXLA functions besides those of *AtEXLA2* in *Arabidopsis thaliana*. *AtEXLA2* was reported to have obvious expression in both the hypocotyl and root; over-expression of *AtEXLA2* resulted in slightly thicker walls in non-rapidly elongating etiolated hypocotyl cells [32]. Phylogenetic tree analysis showed that *GhEXLA1c*, *GhEXLA1f* and *AtEXLA2* were members of the EXLA subfamily (Fig. 1). These results suggested that *GhEXLA1c* and *GhEXLA1f* could participate in the thickening of the cell wall. In addition, it was reported that an expansin-like protein from *Hahella chejuensis* could bind cellulose and enhance cellulase activity [33]. It was implied that the two *EXLA* genes could facilitate cellulose synthesis during the transition; however, the detailed biological functions of EXLAs remain to be assessed in cotton fibre development. More research needs to be conducted in order to understand and make use of EXLAs during cotton transition stages, either in theory or in practice.

## Conclusions

Overall, we successfully performed a genome-scale analysis of the *expansin* family genes in upland cotton with a special emphasis on fibre development. A total of 93 cotton *expansin* genes were obtained. Our analysis has provided information for understanding the cotton expansin superfamily, including gene evolution, gene structure, protein motifs, collinear relationships, *cis*-acting elements and gene expression patterns. Moreover, we obtained the expression patterns of 14 *expansin* genes in relation to cotton fibre development at different stages. Among them, three genes were highly expressed in the initial stage, nine genes had high-level expression during the fast elongation stage, and *GhEXLA1c* and *GhEXLA1f* were preferentially expressed in the transition stage of fibre development. These results lay a foundation for further clarification of the biological functions of *expansin* genes and the molecular mechanisms of many important agricultural traits in cotton, especially in the elongation stage of cotton fibre development.

## Methods

### Plant materials

*Gossypium hirsutum* L. (‘TM-1’) seeds were obtained from the Institute of Cotton Research of the Chinese Academy of Agricultural Sciences (Anyang, China). TM-1 was used as the experimental material in this study. It was planted at the experimental farm (36°06′84.44″N, 114°49′61.5″E) of the Institute of Cotton Research of the Chinese Academy of Agricultural Sciences. To research the expression patterns of *expansin* genes during cotton fibre development, each flower was labelled on the day of flowering, which was considered 0 days post anthesis (DPA). Subsequently, samples were collected at 0, 3, 5, 7, 10, 15, 20, and 30 DPA. The collected bolls were dissected to obtain ovules and fibres. For 0 to 3 DPA samples, we collected ovules, and for 5 to 30 DPA samples, we collected fibres. In addition, we also collected cotton tissue samples at different developmental stages, including roots, hypocotyls, stems, and young leaves at the seedling stage and calycles, petals, pollens and stigmas at the adult stage. The different samples were frozen in liquid nitrogen immediately and stored at − 80 °C in an ultra-low-temperature freezer after harvest.

### Identification and sequence analysis of cotton *expansin* genes

The cotton genomic data were obtained from the Cotton FGD website [34] (https://cottonfgd.org/). Expansin protein sequences of *A. thaliana* were downloaded from TAIR 10 (http:// www.arabidopsis.org/). First, we used the 35 EXPANSIN protein sequences from *A. thaliana* as queries in searches against the *G. hirsutum* genome database [19]. BLASTP with default parameters was used to identify the expansin proteins. After that, we searched the database for homologs using “EXPANSIN” as a keyword; finally, we used 6 previously reported *GhEXPs* as queries to search for other possible *GhEXPs* by BLASTP searches against the cotton genome [30]. Redundant expansin sequences were deleted after a comparison analysis. Then, all candidate expansin protein sequences were submitted to the NCBI CDD (Conserved Domain Database) (https://www.ncbi.nlm.nih.gov/Structure/cdd/wrpsb.cgi) [35], where conserved domains were identified. To ensure a rapid search speed, we performed this work with the Batch Web CD-Search Tool (https://www.ncbi.nlm.nih.gov/Structure/bwrpsb/bwrpsb.cgi) [35], where the maximal number of protein queries per request is 4000, providing adequate processing power for our purposes. We executed the search program using default parameters. The canonical expansin protein contains both conserved domains: DPBB-1 (including the DPBB_1 superfamily) and Pollen_allerg_1 (including the Pollen_allerg_1 superfamily). We acquired the final sequences of the upland cotton expansin family genes for further analysis.

Using the ExPASy online tools [36] (https://www.expasy.org/resources/), we analysed the molecular properties of the identified expansin proteins, which were included to compute the molecular weight (MW) and isoelectric point (pI), and predicted their signal peptide sequences with the SignalP 5.0 server (http://www.cbs.dtu.dk/services/SignalP/). Sequence alignment of the expansin protein sequences was executed in Vector NTI Advance 11 software (version 11.5), followed by searching for the conserved amino acid and domain properties of expansin proteins.

### Phylogenetic tree construction

To analyse phylogenetic relationships, *Arabidopsis thaliana* (*A. thaliana*) expansin protein sequences were downloaded from TAIR (https://www.arabidopsis.org/) and EXPANSIN CENTRAL (http://www.personal.psu.edu/fsl/ExpCentral/). Multiple sequence alignment of the identified cotton expansin and *A. thaliana* expansin proteins was executed in MEGA software (version 6.0) [37], and a phylogenetic tree was constructed with the same software, using the neighbour-joining method. The number of bootstrap replicates was 1000, and the rest of the parameters were set as the defaults.

### Analysis of *expansin* gene structure and motifs

Analysis of gene structure was performed to identify exons, introns, and UTRs. The corresponding GFF data of identified *expansin* gene IDs were extracted from the GFF file named Ghirsutum_gene_model in the new cotton genome data [19] (http://cotton.hzau.edu.cn/EN/download.php), and then the *expansin* GFF data were analysed using the online tool GSDS (version 2.0, http://gsds.cbi.pku.edu.cn/) [38]; the results were saved in SVG image format. Motifs of expansin protein sequences were analysed using the online tool MEME (http://meme-suite.org/index.html) [39]. According to the required file format, we imported the expansin sequences into the online tool. The maximum number of motifs was set to 10, the repeat number was set to 0 or 1, and the remainder of the parameters were set to system defaults. The output draft images of gene structure and motif were further modified with Adobe Illustrator CS3 software (version 13.0.0).

### Chromosomal locations and collinearity relationships of *expansin* genes

We obtained the length of each chromosome from the new genomic data [19], and a file of the lengths of all TM-1 chromosomes was obtained. Then, positional information of the *expansin* gene on the chromosome was extracted from the GFF file, named Ghirsutum_gene_model in the new cotton genome data (http://cotton.hzau.edu.cn/EN/download.php) [19]; thus, a file of positional information of the *expansin* gene was obtained. Afterwards, the two files were submitted to the online tool MG2C (http://mg2c.iask.in/mg2c_v2.0/) for analysis of *expansin* gene location on chromosomes. Collinearity analysis of cotton *expansin* genes was executed by MCScanX software [40], and the visualization of the results was carried out using Circos software [41]. The analysed results were exported in SVG format, and the SVG image was further modified with Adobe Illustrator CS3 software (version 13.0.0).

### Analysis of *cis*-acting regulatory elements in the promoter regions of *expansin* genes

The promoter regions (2000 bp sequence upstream of the transcription start site in the genomic DNA sequence) of the cotton *expansin* genes were identified by searching the *G. hirsutum* genome database (https://cottonfgd.org/) [34], and these promoter sequences were then predicted using PlantCARE (http://bioinformatics.psb.ugent.be/webtools/plantcare/html/) to analyse the *cis*-acting elements [42].

### Transcriptome analysis of cotton *expansin* genes

Total RNA was extracted from ovule and fibre samples. Samples from different stages were used for transcriptome analysis with the Illumina platform, including the ovules at 0 and 3 DPA and fibres at 5, 7, 10, 15, 20, and 30 DPA. For transcriptome sequencing, three biological duplicates were conducted for the experimental samples. The sequencing results of gene expression are shown by FPKM (fragments per kilobase of million mapped reads) values. Expression data FPKM values of *expansin* genes were screened according to the transcriptome results and converted to logFPKM values. Meanwhile, we downloaded RNA-seq data of cotton ovules and fibres from a public database, the Cotton FGD website (https://cottonfgd.org/) [34], including the ovules at − 3, 0, 1, and 3 DPA and fibres at 5, 10, 15, and 20 DPA. The FPKM values were processed as described above. Heatmaps were drawn with the logarithm-transformed values using HemI software (version 1.0) [43].

### RNA isolation and real-time quantitative PCR analysis

The total RNA of samples from different stages was extracted using the RNAprep Pure Kit (for polysaccharide & polyphenolic-rich plants; cat. no. DP441; Tiangen, Beijing, China). The RNA samples were examined using agarose gel electrophoresis, and then the concentration and quality were analysed with a NanoDrop ONE^c^ (Thermo Fisher Scientific, USA). cDNA synthesis was performed on a 2720 thermal cycler (Applied Biosystems, Thermo Fisher Scientific, USA) according to the instructions for the PrimeScript™ II 1st Strand cDNA Synthesis Kit (TaKaRa, Code No. 6210A). All reverse-transcript cDNA samples were diluted 10 times and stored at − 20 °C for the real-time quantification PCR (qRT-PCR) experiment. The design of specific qRT-PCR primers was performed using Beacon Designer software (version 8.0); all the primers are shown in Table S6. qRT-PCRs were performed with the TB Green™ Premix Ex Taq™ II Kit (TaKaRa, Code No. RR820A) and conducted on a QuantStudio™ 5 Real-Time PCR instrument (Applied Biosystems, Thermo Fisher Scientific, USA). *UBQ7* (GenBank No. AY189972) was used as a reference gene to calculate relative expression levels. The data were analysed using the 2^−ΔΔCT^ method [44].

## Supplementary information

**Additional file 1: Table S1**. Identification of *GhEXP* genes in *G. hirsutum*. Additional file 1: **Table S2**. The *GaEXP* genes in *G. arboretum*. **Table S3**. The *GrEXP* genes in *G. raimondii*. **Table S4**. Summary of *cis*-acting elements of *GhEXP* genes. **Table S5**. The number of *cis*-acting elements involved in different biological processes in the promoter region of *GhEXP* genes. **Table S6**. A list of primers used in qRT-PCR experiments.

**Additional file 2: Fig. S1**. Multiple sequence alignment of 93 GhEXP proteins. **Fig. S2**. Analysis of conserved motifs of *GhEXP* genes in cotton. **Fig. S3**. Expression profiles of *GhEXP* genes in cotton ovules and fibres. **Fig. S4**. Quantitative RT-PCR analysis of seven *GhEXP* genes in fibres at different stages and in different tissues. **Fig. S5**. qRT-PCR validation and transcriptome sequencing of 14 *GhEXPs* at different developmental stages. **Fig. S6**. Quantitative RT-PCR analysis of 14 cotton *GhEXP* genes in different tissues.

## Data Availability

All of the data and materials supporting our research findings are contained in the methods section of the manuscript. Details are provided in the attached additional files.

## Abbreviations

DPA: Days post anthesis
*EXPs*: *Expansins*
EXPA: α-expansin
EXPB: β-expansin
EXLA: Expansin-like A
EXLB: Expansin-like B
DPBB: Double-psi beta-barrel
ERE: Ethylene-responsive element
CDS: Coding sequence
