# Supplementary material for: Genome-wide identification of the expansin gene family reveals that expansin genes are involved in fibre cell growth in cotton
Source: BMC Plant Biol. 2020 May 19;20:223. doi: 10.1186/s12870-020-02362-y (PMC7236947; doi:10.1186/s12870-020-02362-y)
Supplement: Supplementary file 1 — Additional file 1: Table S1. Identification of GhEXP genes in G. hirsutum. Additional file 1: Table S2. The GaEXP genes in G. arboretum. Table S3. The GrEXP genes in G. raimondii. Table S4. Summary of cis-acting elements of GhEXP genes. Table S5. The number of cis-acting elements involved in different biological processes in the promoter region of GhEXP genes. Table S6. A list of primers used in qRT-PCR experiments. [file 12870_2020_2362_MOESM1_ESM.zip › Additional file 1.docx]

|  | **Table S1. Identification of *GhEXP* genes in *G. hirsutum*.** | | | | | | | | | | | | | | | | |
| --- | --- | --- | --- | --- | --- | --- | --- | --- | --- | --- | --- | --- | --- | --- | --- | --- | --- |
| **Gene subfamily** | | **Gene ID** | **Gene name** | | **Amino acid residues in length** | **Isoelectric point** | **Molecular weight** | **Signal peptide** | **DPBB_1 conserved domain** | | | | **Pollen_allerg_1 conserved domain** | | | |  |
|  |  |  |  |  |  |  |  |  | **From** | **To** | **E-Value** | **Short name** | **From** | **To** | **E-Value** | **Short name** |  |
| **EXPA** | | Ghir_D13G025630.1 | *GhEXPA8j* | | 249 | 8.36 | 26.86 | 1-23 | 62 | 147 | 2.64E-56 | DPBB_1 | 160 | 231 | 1.23E-32 | Pollen_allerg_1 |  |
|  |  | Ghir_D13G000750.1 | *GhEXPA8i* | | 254 | 8.61 | 27.36 | 1-26 | 67 | 152 | 9.68E-54 | DPBB_1 | 165 | 236 | 3.40E-33 | Pollen_allerg_1 |  |
|  |  | Ghir_D10G019880.1 | *GhEXPA8h* | | 253 | 7.55 | 27.07 | 1-25 | 66 | 151 | 8.83E-53 | DPBB_1 | 164 | 235 | 2.07E-34 | Pollen_allerg_1 |  |
|  |  | Ghir_D05G028410.1 | *GhEXPA8g* | | 252 | 7.53 | 26.92 | 1-24 | 65 | 150 | 1.32E-54 | DPBB_1 | 163 | 234 | 9.23E-34 | Pollen_allerg_1 |  |
|  |  | Ghir_D04G001360.1 | *GhEXPA8f* | | 253 | 8.02 | 27.08 | 1-25 | 66 | 151 | 9.37E-54 | DPBB_1 | 164 | 235 | 1.62E-33 | Pollen_allerg_1 |  |
|  |  | Ghir_A13G024860.1 | *GhEXPA8e* | | 249 | 8.36 | 26.93 | 1-23 | 63 | 147 | 2.31E-55 | DPBB_1 | 160 | 231 | 2.03E-31 | Pollen_allerg_1 |  |
|  |  | Ghir_A13G000460.1 | *GhEXPA8d* | | 254 | 8.41 | 27.28 | 1-26 | 67 | 152 | 1.82E-53 | DPBB_1 | 165 | 236 | 3.24E-34 | Pollen_allerg_1 |  |
|  |  | Ghir_A10G018380.1 | *GhEXPA8c* | | 253 | 7.55 | 26.98 | 1-25 | 66 | 151 | 3.50E-53 | DPBB_1 | 164 | 235 | 1.70E-32 | Pollen_allerg_1 |  |
|  |  | Ghir_A05G041590.1 | *GhEXPA8b* | | 253 | 6.78 | 27.04 | 1-25 | 66 | 151 | 4.72E-54 | DPBB_1 | 164 | 235 | 3.33E-34 | Pollen_allerg_1 |  |
|  |  | Ghir_A05G028400.1 | *GhEXPA8a* | | 253 | 8.07 | 26.88 | 1-25 | 66 | 151 | 2.83E-54 | DPBB_1 | 164 | 235 | 2.10E-33 | Pollen_allerg_1 |  |
|  |  | Ghir_D11G002150.1 | *GhEXPA7e* | | 264 | 9.35 | 29.05 | 1-21 | 28 | 263 | 3.28E-93 | DPBB_1 superfamily | 177 | 244 | 2.06E-20 | Pollen_allerg_1 |  |
|  |  | Ghir_D09G019700.1 | *GhEXPA7d* | | 233 | 5.4 | 25.01 | 1-28 | 27 | 232 | 1.13E-72 | DPBB_1 superfamily | 156 | 213 | 7.32E-13 | Pollen_allerg_1 |  |
|  |  | Ghir_D08G019370.1 | *GhEXPA7c* | | 267 | 8.08 | 29.32 | — | 76 | 162 | 1.38E-44 | DPBB_1 | 180 | 247 | 1.74E-22 | Pollen_allerg_1 |  |
|  |  | Ghir_A11G002140.1 | *GhEXPA7b* | | 264 | 9.36 | 29.23 | 1-29 | 28 | 263 | 1.08E-92 | DPBB_1 superfamily | 177 | 244 | 5.87E-21 | Pollen_allerg_1 |  |
|  |  | Ghir_A09G020220.1 | *GhEXPA7a* | | 265 | 8.75 | 28.70 | 1-28 | 27 | 264 | 7.33E-99 | DPBB_1 superfamily | 178 | 245 | 3.57E-21 | Pollen_allerg_1 |  |
|  |  | Ghir_D09G001390.1 | *GhEXPA5e* | | 366 | 9.41 | 41.53 | — | 185 | 262 | 5.36E-47 | DPBB_1 | 275 | 346 | 5.55E-31 | Pollen_allerg_1 |  |
|  |  | Ghir_D07G022320.1 | *GhEXPA5d* | | 253 | 9.3 | 27.49 | 1-21 | 72 | 149 | 2.59E-49 | DPBB_1 | 162 | 233 | 1.59E-32 | Pollen_allerg_1 |  |
|  |  | Ghir_A10G015740.1 | *GhEXPA5c* | | 244 | 8.82 | 26.00 | 1-20 | 60 | 137 | 1.33E-47 | DPBB_1 | 150 | 220 | 7.04E-31 | Pollen_allerg_1 |  |
|  |  | Ghir_A09G001430.1 | *GhEXPA5b* | | 200 | 9.26 | 21.98 | — | 19 | 96 | 2.83E-48 | DPBB_1 | 109 | 180 | 7.85E-32 | Pollen_allerg_1 |  |
|  |  | Ghir_A07G022240.1 | *GhEXPA5a* | | 253 | 9.4 | 27.52 | 1-21 | 72 | 149 | 1.68E-49 | DPBB_1 | 162 | 233 | 3.53E-31 | Pollen_allerg_1 |  |
|  |  | Ghir_D13G002130.1 | *GhEXPA4r* | | 258 | 9.53 | 28.20 | 1-21 | 67 | 154 | 1.07E-56 | DPBB_1 | 167 | 238 | 1.14E-26 | Pollen_allerg_1 |  |
|  |  | Ghir_D11G036160.1 | *GhEXPA4q* | | 262 | 9.59 | 28.35 | 1-25 | 71 | 158 | 1.03E-55 | DPBB_1 | 171 | 242 | 2.33E-29 | Pollen_allerg_1 |  |
|  |  | Ghir_D10G024930.1 | *GhEXPA4p* | | 258 | 9.57 | 28.09 | 1-21 | 67 | 154 | 7.31E-57 | DPBB_1 | 167 | 238 | 1.35E-28 | Pollen_allerg_1 |  |
|  |  | Ghir_D09G015900.1 | *GhEXPA4o* | | 264 | 9.53 | 28.34 | 1-27 | 73 | 160 | 2.07E-57 | DPBB_1 | 173 | 243 | 7.20E-29 | Pollen_allerg_1 |  |
|  |  | Ghir_D07G010740.1 | *GhEXPA4n* | | 260 | 9.65 | 28.13 | 1-23 | 69 | 156 | 2.01E-56 | DPBB_1 | 169 | 240 | 6.85E-25 | Pollen_allerg_1 |  |
|  |  | Ghir_D05G009460.1 | *GhEXPA4m* | | 224 | 9.73 | 24.36 | — | 33 | 120 | 1.30E-57 | DPBB_1 | 133 | 204 | 4.18E-29 | Pollen_allerg_1 |  |
|  |  | Ghir_D04G013190.1 | *GhEXPA4l* | | 251 | 9.37 | 27.39 | — | 60 | 147 | 6.18E-56 | DPBB_1 | 160 | 231 | 7.61E-29 | Pollen_allerg_1 |  |
|  |  | Ghir_D03G013620.1 | *GhEXPA4k* | | 259 | 9.69 | 28.13 | 1-22 | 68 | 155 | 8.93E-58 | DPBB_1 | 168 | 239 | 7.41E-29 | Pollen_allerg_1 |  |
|  |  | Ghir_D01G023090.1 | *GhEXPA4j* | | 264 | 9.6 | 28.56 | 1-27 | 73 | 160 | 2.20E-56 | DPBB_1 | 173 | 244 | 1.96E-27 | Pollen_allerg_1 |  |
|  |  | Ghir_A13G001850.1 | *GhEXPA4i* | | 258 | 9.43 | 28.02 | 1-21 | 67 | 154 | 5.51E-57 | DPBB_1 | 167 | 238 | 9.52E-26 | Pollen_allerg_1 |  |
|  |  | Ghir_A11G035300.1 | *GhEXPA4h* | | 262 | 9.74 | 28.37 | 1-25 | 71 | 158 | 3.65E-55 | DPBB_1 | 171 | 242 | 1.29E-29 | Pollen_allerg_1 |  |
|  |  | Ghir_A10G022490.1 | *GhEXPA4g* | | 258 | 9.53 | 28.11 | 1-21 | 67 | 154 | 2.57E-57 | DPBB_1 | 167 | 238 | 1.90E-28 | Pollen_allerg_1 |  |
|  |  | Ghir_A10G015240.1 | *GhEXPA4f* | | 258 | 8.36 | 27.94 | 1-30 | 71 | 156 | 1.21E-54 | DPBB_1 | 170 | 240 | 2.38E-33 | Pollen_allerg_1 |  |
|  |  | Ghir_A09G016410.1 | *GhEXPA4e* | | 210 | 9.76 | 22.93 | — | 19 | 106 | 4.95E-57 | DPBB_1 | 119 | 189 | 4.89E-30 | Pollen_allerg_1 |  |
|  |  | Ghir_A07G010680.1 | *GhEXPA4d* | | 252 | 9.75 | 27.76 | — | 61 | 148 | 3.37E-57 | DPBB_1 | 161 | 232 | 3.31E-26 | Pollen_allerg_1 |  |
|  |  | Ghir_A05G009730.1 | *GhEXPA4c* | | 164 | 12.01 | 18.81 | — | 18 | 162 | 6.04E-68 | DPBB_1 superfamily | 73 | 144 | 1.31E-29 | Pollen_allerg_1 |  |
|  |  | Ghir_A04G009030.1 | *GhEXPA4b* | | 264 | 9.19 | 28.50 | 1-27 | 73 | 160 | 9.55E-56 | DPBB_1 | 173 | 244 | 6.68E-29 | Pollen_allerg_1 |  |
|  |  | Ghir_A03G005140.1 | *GhEXPA4a* | | 271 | 9.71 | 29.40 | 1-22 | 68 | 155 | 5.74E-57 | DPBB_1 | 168 | 239 | 2.62E-29 | Pollen_allerg_1 |  |
|  |  | Ghir_D04G004700.1 | *GhEXPA23c* | | 262 | 9.18 | 29.21 | 1-23 | 73 | 158 | 1.67E-51 | DPBB_1 | 171 | 243 | 4.44E-27 | Pollen_allerg_1 |  |
|  |  | Ghir_A05G038370.1 | *GhEXPA23b* | | 262 | 9.19 | 29.05 | 1-23 | 73 | 158 | 1.08E-51 | DPBB_1 | 171 | 243 | 1.30E-25 | Pollen_allerg_1 |  |
|  |  | Ghir_A05G038360.1 | *GhEXPA23a* | | 262 | 9.25 | 29.18 | 1-22 | 73 | 158 | 5.17E-52 | DPBB_1 | 171 | 243 | 2.90E-26 | Pollen_allerg_1 |  |
|  |  | Ghir_D12G001280.1 | *GhEXPA20b* | | 207 | 8.38 | 22.67 | 1-19 | 64 | 150 | 3.22E-15 | DPBB_1 | 163 | 207 | 2.66E-13 | Pollen_allerg_1 superfamily |  |
|  |  | Ghir_A12G001280.1 | *GhEXPA20a* | | 253 | 8.57 | 27.78 | 1-23 | 4 | 252 | 2.79E-93 | DPBB_1 superfamily | 163 | 234 | 1.81E-25 | Pollen_allerg_1 |  |
|  |  | Ghir_D10G012330.1 | *GhEXPA2* | | 258 | 8.05 | 27.96 | 1-23 | 71 | 156 | 1.50E-54 | DPBB_1 | 170 | 240 | 7.10E-31 | Pollen_allerg_1 |  |
|  |  | Ghir_D12G019140.1 | *GhEXPA1e* | | 230 | 9.26 | 24.88 | 1-20 | 1 | 229 | 8.28E-127 | DPBB_1 superfamily | 140 | 211 | 1.62E-34 | Pollen_allerg_1 |  |
|  |  | Ghir_D05G018790.1 | *GhEXPA1d* | | 255 | 9.19 | 27.36 | 1-23 | 64 | 152 | 1.42E-54 | DPBB_1 | 165 | 235 | 3.42E-29 | Pollen_allerg_1 |  |
|  |  | Ghir_A12G018930.1 | *GhEXPA1c* | | 248 | 9.19 | 26.62 | 1-20 | 60 | 145 | 7.14E-54 | DPBB_1 | 158 | 229 | 2.37E-34 | Pollen_allerg_1 |  |
|  |  | Ghir_A06G000150.1 | *GhEXPA1b* | | 255 | 9.15 | 27.38 | 1-23 | 64 | 152 | 1.41E-54 | DPBB_1 | 165 | 235 | 1.72E-28 | Pollen_allerg_1 |  |
|  |  | Ghir_A05G018780.1 | *GhEXPA1a* | | 289 | 9.19 | 31.29 | — | 98 | 186 | 5.47E-54 | DPBB_1 | 199 | 269 | 8.62E-29 | Pollen_allerg_1 |  |
|  |  | Ghir_D08G013800.1 | *GhEXPA17d* | | 254 | 9.31 | 28.01 | 1-23 | 63 | 150 | 1.45E-50 | DPBB_1 | 163 | 234 | 4.11E-30 | Pollen_allerg_1 |  |
|  |  | Ghir_D07G014300.1 | *GhEXPA17c* | | 235 | 9.71 | 25.44 | 1-25 | 43 | 131 | 3.08E-49 | DPBB_1 | 144 | 215 | 3.32E-26 | Pollen_allerg_1 |  |
|  |  | Ghir_A11G011410.1 | *GhEXPA17b* | | 258 | 9.43 | 28.42 | — | 66 | 154 | 2.68E-54 | DPBB_1 | 167 | 238 | 2.04E-29 | Pollen_allerg_1 |  |
|  |  | Ghir_A07G014060.1 | *GhEXPA17a* | | 258 | 9.7 | 27.92 | — | 66 | 154 | 3.20E-50 | DPBB_1 | 167 | 238 | 1.29E-26 | Pollen_allerg_1 |  |
|  |  | Ghir_D13G007870.1 | *GhEXPA15g* | | 248 | 9.3 | 26.33 | 1-17 | 60 | 145 | 3.09E-54 | DPBB_1 | 158 | 229 | 1.69E-34 | Pollen_allerg_1 |  |
|  |  | Ghir_D11G026180.1 | *GhEXPA15f* | | 247 | 9.3 | 26.35 | 1-20 | 60 | 145 | 2.51E-52 | DPBB_1 | 158 | 229 | 6.76E-32 | Pollen_allerg_1 |  |
|  |  | Ghir_D05G031310.1 | *GhEXPA15e* | | 246 | 9.04 | 26.42 | 1-22 | 62 | 143 | 4.78E-45 | DPBB_1 | 156 | 227 | 1.31E-32 | Pollen_allerg_1 |  |
|  |  | Ghir_A13G007620.1 | *GhEXPA15d* | | 202 | 9.47 | 21.57 | 1-17 | 1 | 201 | 4.54E-90 | DPBB_1 superfamily | 112 | 183 | 2.13E-33 | Pollen_allerg_1 |  |
|  |  | Ghir_A11G026010.1 | *GhEXPA15c* | | 247 | 9.32 | 26.39 | 1-20 | 60 | 145 | 7.84E-53 | DPBB_1 | 158 | 229 | 5.63E-32 | Pollen_allerg_1 |  |
|  |  | Ghir_A05G031460.1 | *GhEXPA15b* | | 262 | 8.97 | 28.18 |  | 78 | 159 | 2.75E-45 | DPBB_1 | 172 | 243 | 7.11E-34 | Pollen_allerg_1 |  |
|  |  | Ghir_A03G011510.1 | *GhEXPA15a* | | 250 | 9.38 | 26.85 | 1-22 | 62 | 147 | 3.62E-53 | DPBB_1 | 160 | 230 | 1.37E-34 | Pollen_allerg_1 |  |
|  |  | Ghir_D07G008140.1 | *GhEXPA13b* | | 314 | 8.38 | 33.99 | — | 87 | 312 | 1.56E-92 | DPBB_1 superfamily | 223 | 294 | 2.50E-28 | Pollen_allerg_1 |  |
|  |  | Ghir_A07G008050.1 | *GhEXPA13a* | | 293 | 8.35 | 31.64 | — | 87 | 293 | 7.34E-81 | DPBB_1 superfamily | 223 | 293 | 2.96E-28 | Pollen_allerg_1 |  |
|  |  | Ghir_D01G005810.1 | *GhEXPA12b* | | 211 | 10.13 | 23.50 | — | 6 | 210 | 3.43E-95 | DPBB_1 superfamily | 119 | 190 | 6.52E-26 | Pollen_allerg_1 |  |
|  |  | Ghir_A01G005570.1 | *GhEXPA12a* | | 256 | 9.9 | 28.40 | 1-27 | 30 | 255 | 1.23E-100 | DPBB_1 superfamily | 164 | 235 | 1.26E-23 | Pollen_allerg_1 |  |
|  |  | Ghir_D05G025990.1 | *GhEXPA11b* | | 255 | 8.88 | 27.45 | 1-23 | 64 | 152 | 4.53E-51 | DPBB_1 | 165 | 235 | 1.61E-29 | Pollen_allerg_1 |  |
|  |  | Ghir_A05G025950.1 | *GhEXPA11a* | | 258 | 9.07 | 27.89 | 1-23 | 64 | 152 | 9.86E-52 | DPBB_1 | 168 | 238 | 4.14E-29 | Pollen_allerg_1 |  |
| **EXLA** | | Ghir_D04G018220.1 | *GhEXLA1f* | | 259 | 8.59 | 28.13 | 1-18 | 3 | 243 | 2.01E-73 | DPBB_1 superfamily | 147 | 215 | 1.90E-22 | Pollen_allerg_1 |  |
|  |  | Ghir_D02G024020.1 | *GhEXLA1e* | | 260 | 8.24 | 28.21 | 1-20 | 1 | 245 | 1.09E-80 | DPBB_1 superfamily | 148 | 222 | 1.97E-23 | Pollen_allerg_1 |  |
|  |  | Ghir_D01G018080.1 | *GhEXLA1d* | | 259 | 8.26 | 28.01 | 1-17 | 25 | 243 | 1.67E-73 | DPBB_1 superfamily | 146 | 214 | 2.19E-22 | Pollen_allerg_1 |  |
|  |  | Ghir_A04G013790.1 | *GhEXLA1c* | | 259 | 8.59 | 28.10 | 1-18 | 3 | 243 | 2.22E-73 | DPBB_1 superfamily | 147 | 215 | 2.16E-22 | Pollen_allerg_1 |  |
|  |  | Ghir_A03G022570.1 | *GhEXLA1b* | | 295 | 8.92 | 32.41 | — | 36 | 280 | 6.11E-79 | DPBB_1 superfamily | 183 | 257 | 4.12E-23 | Pollen_allerg_1 |  |
|  |  | Ghir_A01G016520.1 | *GhEXLA1a* | | 277 | 8.26 | 30.15 | 1-35 | 43 | 261 | 1.45E-72 | DPBB_1 superfamily | 164 | 232 | 5.03E-23 | Pollen_allerg_1 |  |
|  |  | Ghir_D11G011380.1 | *GhEXLA17e* | | 125 | 11.2 | 14.29 | — | 1 | 124 | 5.99E-60 | DPBB_1 superfamily | 34 | 105 | 7.76E-29 | Pollen_allerg_1 |  |
| **EXPB** | | Ghir_A12G014930.1 | *GhEXPB3a* | | 267 | 8.71 | 29.12 | 1-27 | 44 | 264 | 1.61E-43 | DPBB_1 superfamily | 171 | 246 | 2.44E-29 | Pollen_allerg_1 |  |
|  |  | Ghir_D12G015130.1 | *GhEXPB3b* | | 206 | 7.49 | 22.34 | 1-24 | 58 | 137 | 4.45E-16 | DPBB_1 | 151 | 182 | 2.62E-09 | Pollen_allerg_1 superfamily |  |
|  |  | Ghir_D12G010410.1 | *GhEXPB2d* | | 238 | 5.9 | 25.33 | — | 61 | 140 | 3.36E-14 | DPBB_1 | 154 | 232 | 5.65E-32 | Pollen_allerg_1 |  |
|  |  | Ghir_D08G015350.1 | *GhEXPB2c* | | 273 | 5.03 | 28.61 | — | 44 | 270 | 4.81E-40 | DPBB_1 superfamily | 175 | 252 | 8.15E-31 | Pollen_allerg_1 |  |
|  |  | Ghir_A12G010250.1 | *GhEXPB2b* | | 283 | 5.82 | 30.20 | — | 84 | 127 | 4.92E-09 | DPBB_1 | 184 | 262 | 8.12E-30 | Pollen_allerg_1 |  |
|  |  | Ghir_A08G014510.1 | *GhEXPB2a* | | 273 | 5.19 | 28.60 | — | 50 | 270 | 8.88E-39 | DPBB_1 superfamily | 175 | 252 | 1.98E-31 | Pollen_allerg_1 |  |
|  |  | Ghir_D02G018750.1 | *GhEXPB1b* | | 275 | 8.98 | 30.25 | 1-34 | 52 | 273 | 2.45E-41 | DPBB_1 superfamily | 180 | 255 | 2.41E-30 | Pollen_allerg_1 |  |
|  |  | Ghir_A03G017480.1 | *GhEXPB1a* | | 256 | 7.59 | 28.02 | 1-26 | 44 | 254 | 2.33E-34 | DPBB_1 superfamily | 161 | 236 | 2.76E-30 | Pollen_allerg_1 |  |
| **EXLB** | | Ghir_D12G022410.1 | *GhEXLB1l* | 249 | | 4.65 | 27.12 | 1-21 | 24 | 248 | 1.48E-87 | DPBB_1 superfamily | 150 | 217 | 1.74E-13 | Pollen_allerg_1 |  |
|  |  | Ghir_D08G018040.1 | *GhEXLB1k* | 250 | | 6.12 | 27.57 | 1-24 | 3 | 249 | 1.90E-155 | DPBB_1 superfamily | 153 | 227 | 6.93E-19 | Pollen_allerg_1 |  |
|  |  | Ghir_D08G018030.1 | *GhEXLB1j* | 248 | | 8.96 | 27.55 | 1-25 | 9 | 247 | 1.95E-130 | DPBB_1 superfamily | 155 | 220 | 1.12E-13 | Pollen_allerg_1 |  |
|  |  | Ghir_D08G018020.1 | *GhEXLB1i* | 249 | | 6.06 | 27.36 | 1-25 | 9 | 249 | 8.91E-135 | DPBB_1 superfamily | 154 | 219 | 8.84E-16 | Pollen_allerg_1 |  |
|  |  | Ghir_D08G017670.1 | *GhEXLB1h* | 256 | | 4.66 | 27.90 | 1-24 | 31 | 254 | 1.58E-87 | DPBB_1 superfamily | 157 | 224 | 1.32E-13 | Pollen_allerg_1 |  |
|  |  | Ghir_D03G018210.1 | *GhEXLB1g* | 225 | | 6.79 | 24.97 | 1-24 | 3 | 224 | 8.72E-127 | DPBB_1 superfamily | 128 | 193 | 1.13E-20 | Pollen_allerg_1 |  |
|  |  | Ghir_A12G022440.1 | *GhEXLB1f* | 249 | | 4.65 | 27.08 | 1-21 | 24 | 248 | 1.06E-87 | DPBB_1 superfamily | 150 | 217 | 9.47E-14 | Pollen_allerg_1 |  |
|  |  | Ghir_A08G017220.1 | *GhEXLB1e* | 250 | | 6.79 | 27.64 | 1-24 | 3 | 249 | 1.61E-155 | DPBB_1 superfamily | 153 | 227 | 1.47E-19 | Pollen_allerg_1 |  |
|  |  | Ghir_A08G017190.1 | *GhEXLB1d* | 248 | | 9 | 27.67 | — | 9 | 247 | 6.42E-133 | DPBB_1 superfamily | 155 | 216 | 3.15E-14 | Pollen_allerg_1 |  |
|  |  | Ghir_A08G017180.1 | *GhEXLB1c* | 235 | | 6.4 | 25.92 | 1-25 | 9 | 235 | 8.21E-124 | DPBB_1 superfamily | 154 | 227 | 4.77E-16 | Pollen_allerg_1 |  |
|  |  | Ghir_A08G016840.1 | *GhEXLB1b* | 256 | | 4.81 | 27.81 | 1-24 | 31 | 254 | 5.90E-89 | DPBB_1 superfamily | 157 | 224 | 2.08E-13 | Pollen_allerg_1 |  |
|  |  | Ghir_A03G000980.1 | *GhEXLB1a* | 236 | | 5.64 | 26.16 | — | 1 | 235 | 5.32E-148 | DPBB_1 superfamily | 139 | 213 | 1.45E-19 | Pollen_allerg_1 |  |
| Note: Gene names are given according to the nomenclature guidelines in the literature. | | | | | | | | | | | | | | | | | |

| **Table S4. Summary of *cis*-acting elements of *GhEXP* genes** | | | | | | | |
| --- | --- | --- | --- | --- | --- | --- | --- |
| **Gene**  **name** | **Light-responsive elements** | **Development-related elements** | **Hormone-responsive elements** | **Environmental stress-related elements** | **Promoter-related elements** | **Site-binding-related elements** | **Others** |
| **Total** | **1268** | **144** | **779** | **409** | **9416** | **81** | **3103** |
| *GhEXPA8j* | 14 | 7 | 4 | 1 | 102 | 1 | 49 |
| *GhEXPA8i* | 11 | 4 | 10 | 4 | 68 | 0 | 28 |
| *GhEXPA8h* | 11 | 0 | 7 | 6 | 194 | 2 | 54 |
| *GhEXPA8g* | 20 | 6 | 9 | 4 | 118 | 2 | 19 |
| *GhEXPA8f* | 11 | 1 | 5 | 4 | 173 | 4 | 32 |
| *GhEXPA8e* | 15 | 1 | 7 | 3 | 137 | 0 | 31 |
| *GhEXPA8d* | 16 | 1 | 10 | 5 | 75 | 0 | 39 |
| *GhEXPA8c* | 16 | 0 | 5 | 4 | 122 | 0 | 35 |
| *GhEXPA8b* | 13 | 0 | 8 | 3 | 85 | 1 | 22 |
| *GhEXPA8a* | 17 | 3 | 10 | 1 | 91 | 1 | 23 |
| *GhEXPA7e* | 20 | 0 | 12 | 9 | 115 | 1 | 39 |
| *GhEXPA7d* | 13 | 2 | 18 | 2 | 96 | 0 | 38 |
| *GhEXPA7c* | 14 | 0 | 16 | 3 | 95 | 1 | 36 |
| *GhEXPA7b* | 14 | 0 | 4 | 4 | 128 | 0 | 32 |
| *GhEXPA7a* | 14 | 0 | 12 | 6 | 110 | 1 | 38 |
| *GhEXPA5e* | 8 | 1 | 12 | 7 | 125 | 1 | 45 |
| *GhEXPA5d* | 12 | 2 | 9 | 2 | 67 | 0 | 44 |
| *GhEXPA5c* | 17 | 3 | 3 | 3 | 127 | 1 | 43 |
| *GhEXPA5b* | 13 | 3 | 1 | 14 | 125 | 0 | 29 |
| *GhEXPA5a* | 12 | 1 | 9 | 1 | 101 | 0 | 23 |
| *GhEXPA4r* | 18 | 2 | 11 | 5 | 115 | 1 | 42 |
| *GhEXPA4q* | 11 | 0 | 3 | 3 | 106 | 1 | 28 |
| *GhEXPA4p* | 18 | 1 | 12 | 3 | 112 | 2 | 32 |
| *GhEXPA4o* | 13 | 2 | 3 | 6 | 89 | 1 | 41 |
| *GhEXPA4n* | 15 | 0 | 15 | 1 | 98 | 1 | 36 |
| *GhEXPA4m* | 11 | 2 | 18 | 6 | 97 | 0 | 28 |
| *GhEXPA4l* | 10 | 2 | 3 | 7 | 100 | 0 | 36 |
| *GhEXPA4k* | 6 | 1 | 11 | 10 | 105 | 2 | 45 |
| *GhEXPA4j* | 13 | 0 | 6 | 6 | 93 | 1 | 41 |
| *GhEXPA4i* | 16 | 4 | 10 | 5 | 71 | 0 | 36 |
| *GhEXPA4h* | 8 | 2 | 8 | 1 | 84 | 0 | 27 |
| *GhEXPA4g* | 12 | 3 | 8 | 7 | 109 | 3 | 29 |
| *GhEXPA4f* | 6 | 0 | 7 | 1 | 68 | 0 | 39 |
| *GhEXPA4e* | 25 | 2 | 20 | 1 | 113 | 2 | 32 |
| *GhEXPA4d* | 25 | 0 | 13 | 4 | 117 | 2 | 17 |
| *GhEXPA4c* | 10 | 2 | 7 | 7 | 106 | 0 | 54 |
| *GhEXPA4b* | 10 | 3 | 1 | 5 | 68 | 0 | 29 |
| *GhEXPA4a* | 16 | 0 | 6 | 2 | 105 | 1 | 31 |
| *GhEXPA23c* | 4 | 3 | 1 | 0 | 70 | 0 | 6 |
| *GhEXPA23b* | 17 | 2 | 4 | 8 | 90 | 0 | 31 |
| *GhEXPA23a* | 4 | 1 | 4 | 9 | 92 | 1 | 24 |
| *GhEXPA20b* | 9 | 3 | 12 | 1 | 117 | 2 | 23 |
| *GhEXPA20a* | 13 | 7 | 9 | 4 | 116 | 0 | 34 |
| *GhEXPA2* | 14 | 0 | 2 | 1 | 137 | 1 | 36 |
| *GhEXPA1e* | 11 | 3 | 4 | 3 | 122 | 1 | 36 |
| *GhEXPA1d* | 7 | 1 | 9 | 3 | 102 | 3 | 30 |
| *GhEXPA1c* | 11 | 1 | 10 | 3 | 111 | 0 | 38 |
| *GhEXPA1b* | 15 | 3 | 13 | 7 | 71 | 2 | 32 |
| *GhEXPA1a* | 14 | 3 | 14 | 3 | 60 | 0 | 31 |
| *GhEXPA17d* | 13 | 0 | 16 | 7 | 72 | 1 | 31 |
| *GhEXPA17c* | 11 | 1 | 6 | 5 | 121 | 1 | 29 |
| *GhEXPA17b* | 13 | 0 | 10 | 6 | 76 | 0 | 38 |
| *GhEXPA17a* | 19 | 4 | 10 | 6 | 115 | 2 | 24 |
| *GhEXPA15g* | 11 | 1 | 4 | 6 | 178 | 0 | 51 |
| *GhEXPA15f* | 17 | 0 | 11 | 2 | 109 | 0 | 24 |
| *GhEXPA15e* | 19 | 2 | 7 | 7 | 101 | 2 | 21 |
| *GhEXPA15d* | 19 | 0 | 3 | 3 | 179 | 0 | 29 |
| *GhEXPA15c* | 17 | 2 | 9 | 4 | 88 | 3 | 24 |
| *GhEXPA15b* | 15 | 1 | 5 | 2 | 100 | 0 | 21 |
| *GhEXPA15a* | 26 | 0 | 11 | 5 | 112 | 1 | 40 |
| *GhEXPA13b* | 11 | 2 | 5 | 7 | 55 | 1 | 44 |
| *GhEXPA13a* | 12 | 2 | 14 | 3 | 95 | 3 | 34 |
| *GhEXPA12b* | 16 | 0 | 5 | 5 | 109 | 0 | 30 |
| *GhEXPA12a* | 9 | 0 | 20 | 4 | 46 | 0 | 45 |
| *GhEXPA11b* | 12 | 2 | 9 | 0 | 90 | 1 | 36 |
| *GhEXPA11a* | 11 | 1 | 5 | 3 | 73 | 1 | 32 |
| *GhEXLA1f* | 9 | 0 | 6 | 4 | 88 | 1 | 28 |
| *GhEXLA1e* | 9 | 2 | 2 | 8 | 84 | 0 | 42 |
| *GhEXLA1d* | 39 | 1 | 11 | 3 | 116 | 2 | 40 |
| *GhEXLA1c* | 9 | 4 | 4 | 5 | 73 | 1 | 35 |
| *GhEXLA1b* | 16 | 0 | 15 | 4 | 97 | 0 | 32 |
| *GhEXLA1a* | 4 | 3 | 6 | 6 | 53 | 0 | 40 |
| *GhEXLA17e* | 8 | 3 | 1 | 7 | 89 | 0 | 36 |
| *GhEXPB3a* | 23 | 1 | 11 | 3 | 87 | 2 | 25 |
| *GhEXPB3b* | 12 | 1 | 14 | 4 | 94 | 1 | 33 |
| *GhEXPB2d* | 11 | 0 | 8 | 5 | 118 | 1 | 48 |
| *GhEXPB2c* | 24 | 2 | 11 | 6 | 62 | 0 | 39 |
| *GhEXPB2b* | 7 | 2 | 12 | 5 | 81 | 1 | 29 |
| *GhEXPB2a* | 10 | 0 | 8 | 4 | 79 | 0 | 35 |
| *GhEXPB1b* | 22 | 1 | 15 | 1 | 100 | 1 | 29 |
| *GhEXPB1a* | 21 | 1 | 13 | 2 | 109 | 2 | 19 |
| *GhEXLB1l* | 7 | 1 | 6 | 10 | 81 | 0 | 48 |
| *GhEXLB1k* | 13 | 4 | 3 | 6 | 73 | 0 | 28 |
| *GhEXLB1j* | 25 | 2 | 11 | 6 | 96 | 3 | 31 |
| *GhEXLB1i* | 4 | 0 | 5 | 5 | 98 | 2 | 26 |
| *GhEXLB1h* | 14 | 0 | 10 | 5 | 145 | 1 | 28 |
| *GhEXLB1g* | 8 | 3 | 9 | 7 | 67 | 0 | 38 |
| *GhEXLB1f* | 10 | 0 | 8 | 4 | 129 | 2 | 26 |
| *GhEXLB1e* | 14 | 5 | 6 | 4 | 107 | 0 | 32 |
| *GhEXLB1d* | 17 | 0 | 2 | 3 | 125 | 0 | 37 |
| *GhEXLB1c* | 9 | 0 | 2 | 0 | 128 | 0 | 31 |
| *GhEXLB1b* | 15 | 1 | 7 | 4 | 109 | 2 | 41 |
| *GhEXLB1a* | 14 | 1 | 8 | 5 | 111 | 0 | 31 |

| **Table S6. A list of primers used in qRT-PCR experiments.** | | |
| --- | --- | --- |
| **Gene names** | **Primer names** | **Sequences (5'---3')** |
| *GhEXPA4o* | QF-15900 | AAGGCTATGGCGTGAATA |
|  | QR-15900 | GTGGCAGTGATAATAATGGAA |
| *GhEXPA1a* | QF-18780 | CTGCCTTGTTCAATAATGGA |
|  | QR-18780 | GTAATGGTCACGGATGTTC |
| *GhEXPA8h* | qF1_1861 | CACTGCAGCACTTAGCATTGCC |
|  | qR1_1861 | AGCAGGCTCAGCCAAATCGAAA |
| *GhEXPA4a* | qF1-0359 | ATGGCTTCCGTTACTTCA |
|  | qR1-0359 | AATGATAATGATTGACCAACCA |
| *GhEXPA13a* | qF1-0664 | GGCGTTGTAATCCTCCTA |
|  | qR1-0664 | AACATTGCTGATGAGAACTG |
| *GhEXPA8g* | qF1-2650 | TACTTCAACTTGGTCCTCAT |
|  | qR1-2650 | CTTGTGGTGACTTGGAATG |
| *GhEXPA8a* | qF1_2385 | ACTGCAACCAACTTCTGTCCCC |
|  | qR1_2385 | TGAAATGGGGACGATTCCAGCC |
| *GhEXPA4q* | QF-36160 | CTTAGTTGTGGTGCTTGT |
|  | QR-36160 | CGTTGACCGTGAATCTTAT |
| *GhEXPA8f* | qF1-1924 | CTTCTCTTCTGCTCTTCCT |
|  | qR1-1924 | CATCACCACCACCATAGA |
| *GhEXPA4n* | qF1-0974 | GGATTCCGATACTTCAACTTAG |
|  | qR1-0974 | ACTGACCAACTAGCACAA |
| *GhEXPA2* | qF1_1145 | GTGGGAGGGGCAGGGGATATAA |
|  | qR1_1145 | GAGAGGCTTTGGCCGTTAAGGT |
| *GhEXPA4f* | qF1_2323 | CGGCATTCTTGCGGATAGCAGA |
|  | qR1_2323 | TTATATCCCCTGCCCCTCCCAC |
| *GhEXLA1c* | QF-13790 | TCTGCTACTGCTTGTGAT |
|  | QR-13790 | CTAATGCCAAGGAACCATATC |
| *GhEXLA1f* | QF-18220 | TTGTGATCGTTGCCTGTA |
|  | QR-18220 | CTAATGCCAAGGAACCATATC |
| *GhEXPA12b* | qF-5810 | TACATGGCTGCCTATGAATA |
|  | qR-5810 | TGTCGTGTTGAAGCAAAG |
| *GhEXLB1a* | qF-0980 | GATGGCACAATCGGACAAAGCG |
|  | qR-0980 | AATCTCAAAGTGACCGCTCCCG |
| *GhEXPA11a* | qF-25950 | GGTATCTACAGAGGCAGAAT |
|  | qR-25950 | CCTTGATGGACACAGACT |
| *GhEXPB2a* | qF-14510 | TTCGATTTAAGTGGTACTTCTTT |
|  | qR-14510 | CGTTCCAGGATAGTTACATTC |
| *GhEXPA17d* | qF-13800 | CTCAAGAGTAGATACATTACCATC |
|  | qR-13800 | GGAGGATTACACCAACCA |
| *GhEXPB2c* | qF-15350 | TTCGATTTAAGTGGTACTTCTTT |
|  | qR-15350 | CGTTCCAGGATAGTTACATTC |
| *GhEXLB1h* | qF-17670 | CATTCACGAGCAGCATAC |
|  | qR-17670 | GCGGTAGAGGTCAGATAC |
| *GhUBQ7* | GhUBQ7-qF | GAAGGCATTCCACCTGACCAAC |
|  | GhUBQ7-qR | CTTGACCTTCTTCTTCTTGTGCTTG |
