# Supplementary material for: Genome-wide identification of the expansin gene family reveals that expansin genes are involved in fibre cell growth in cotton
Source: BMC Plant Biol. 2020 May 19;20:223. doi: 10.1186/s12870-020-02362-y (PMC7236947; doi:10.1186/s12870-020-02362-y)

**Figure S1.** **Multiple sequence alignment of 93 GhEXP proteins**. The 93 deduced expansin proteins were aligned using Vector NTI software. The signal peptide, conserved domain, conserved amino acid sites, and EXLA extension are clearly marked with black lines or arrows.


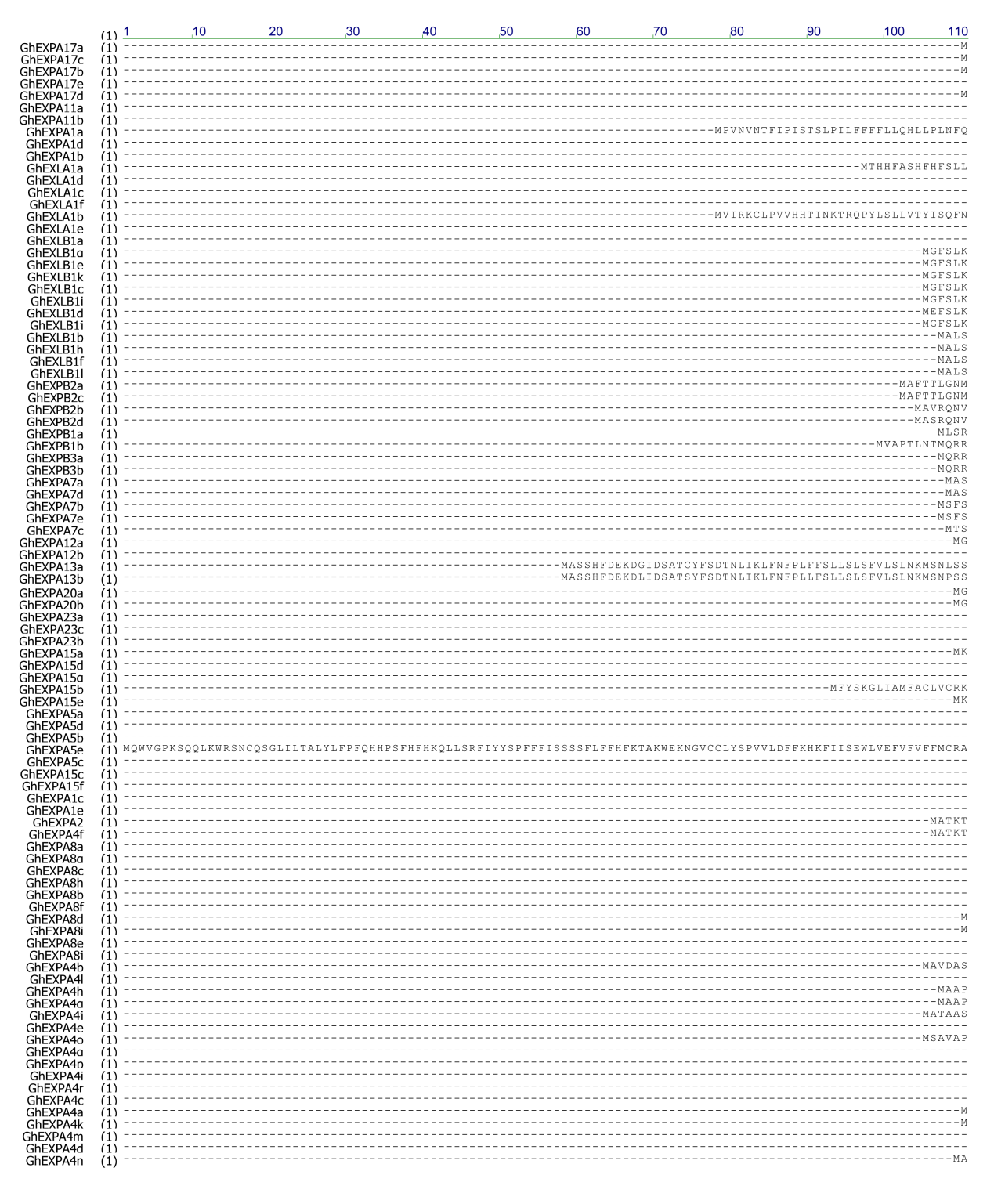
**
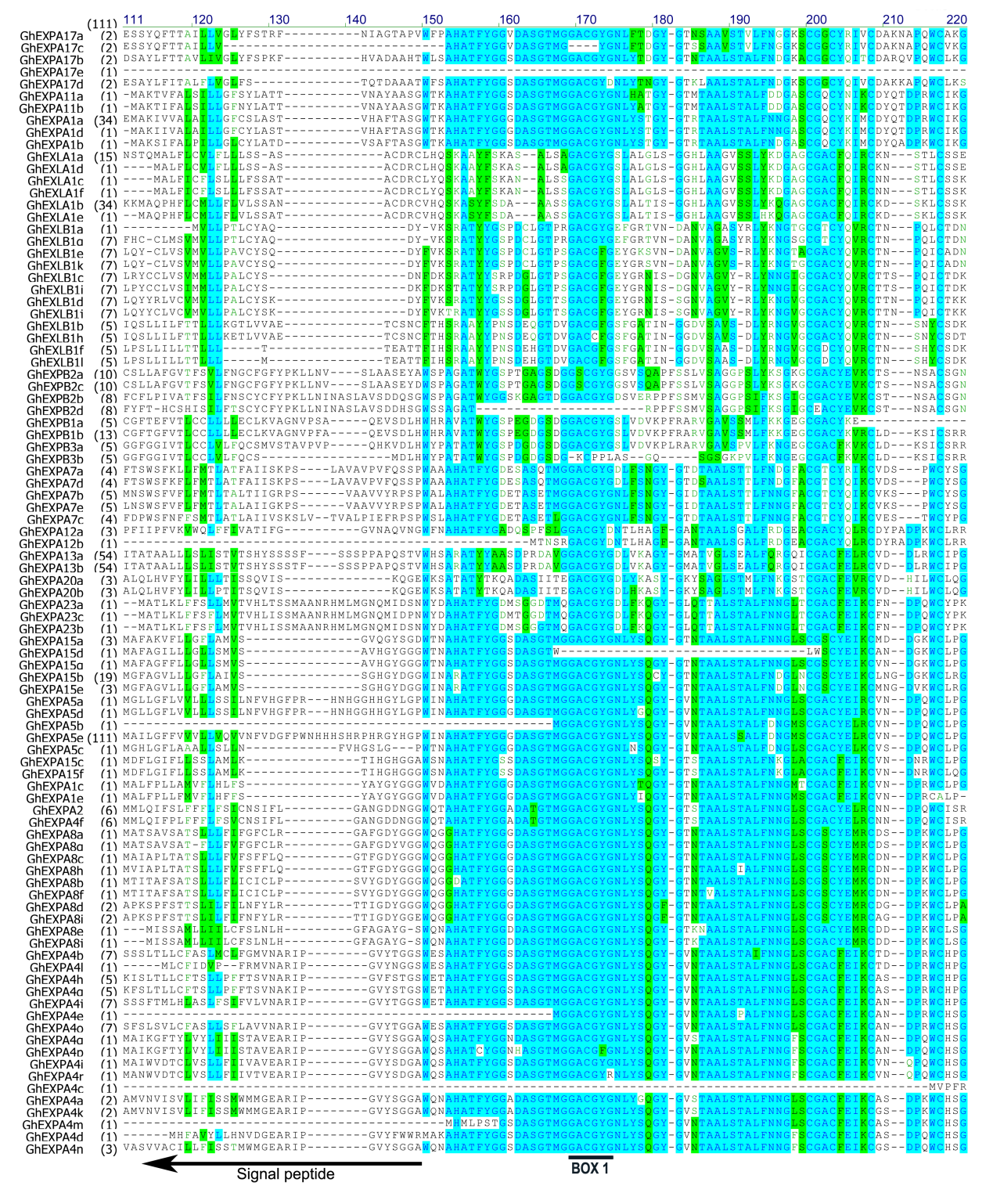
**
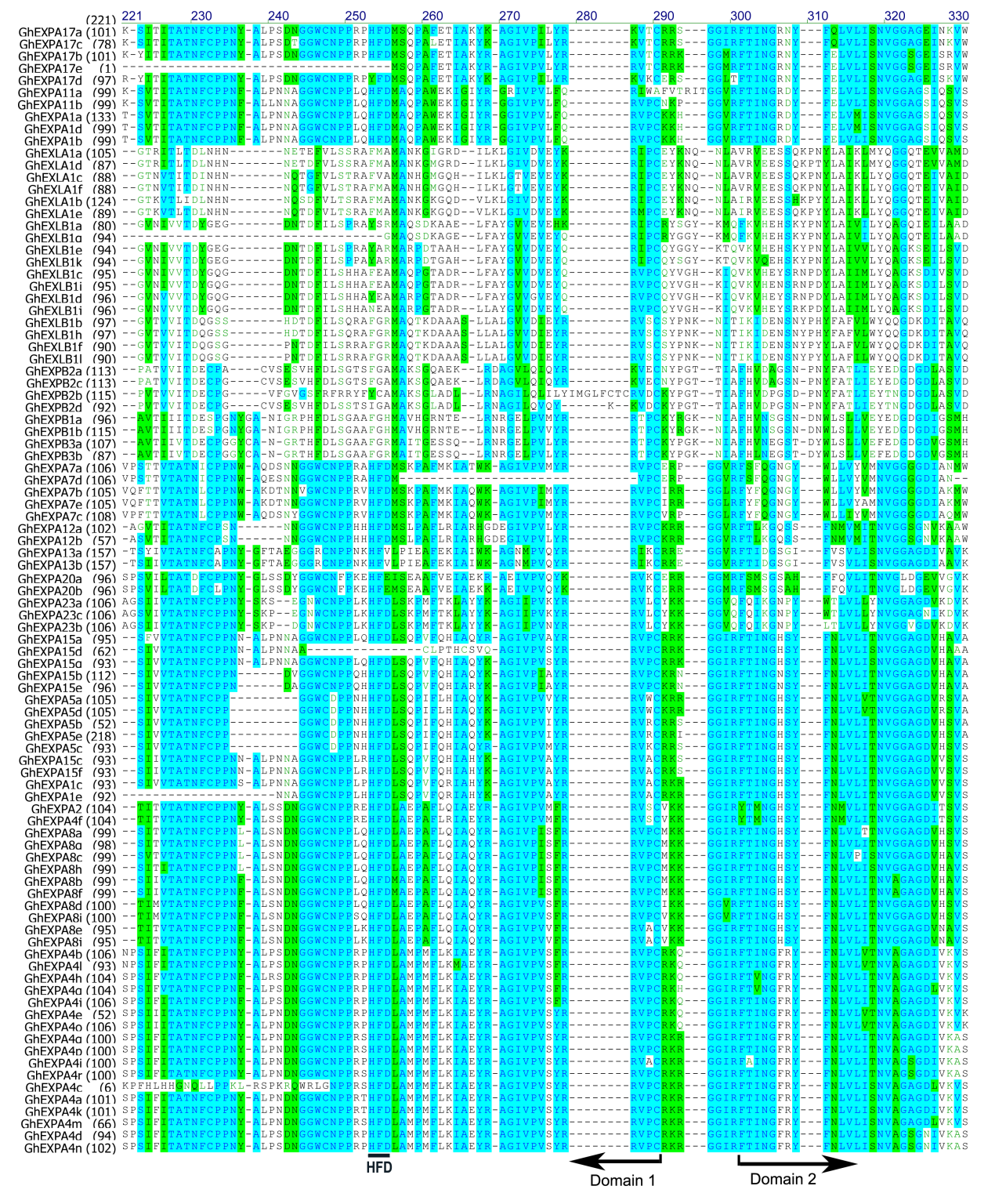

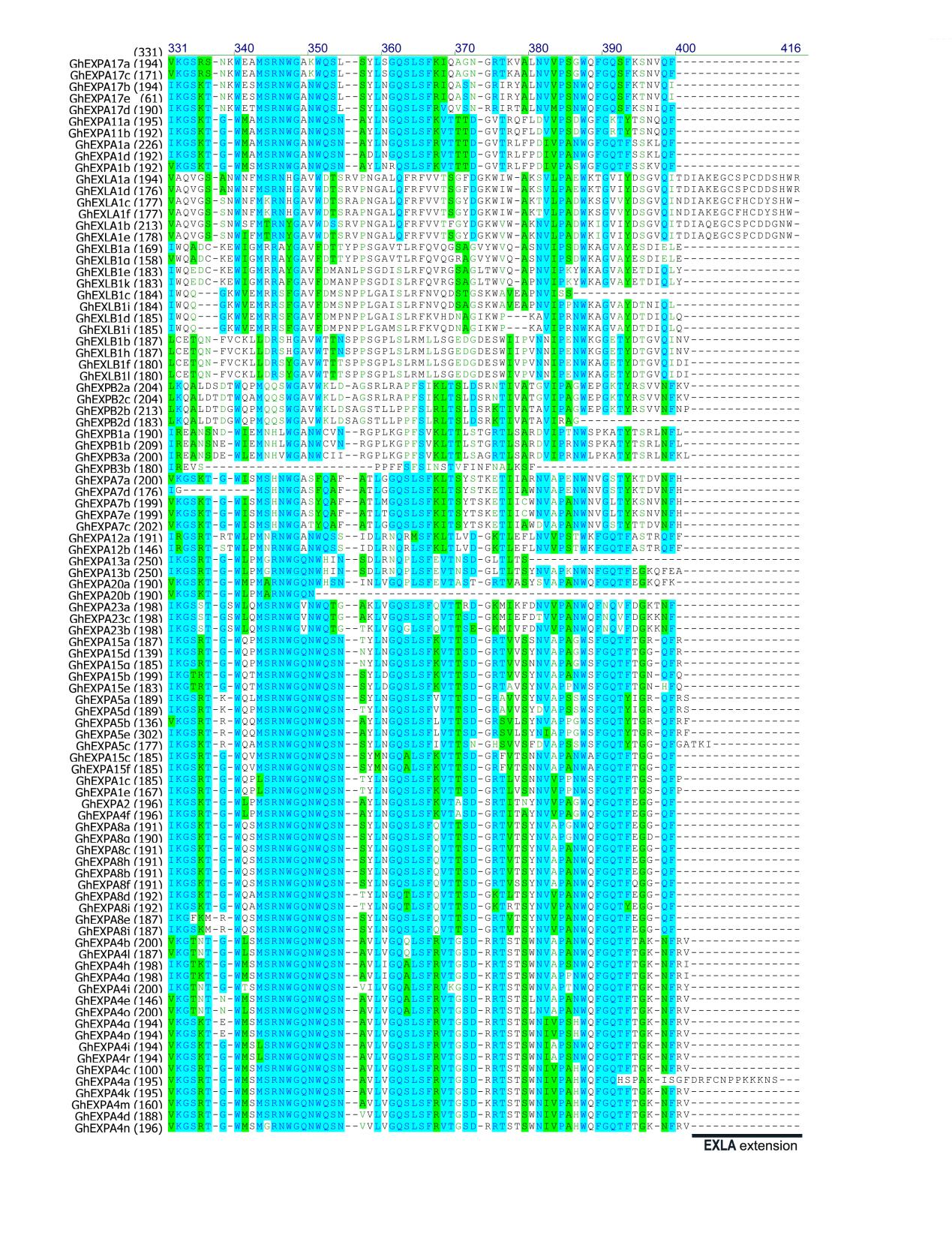


**Figure S2. Analysis of conserved motifs of GhEXP genes in cotton.**


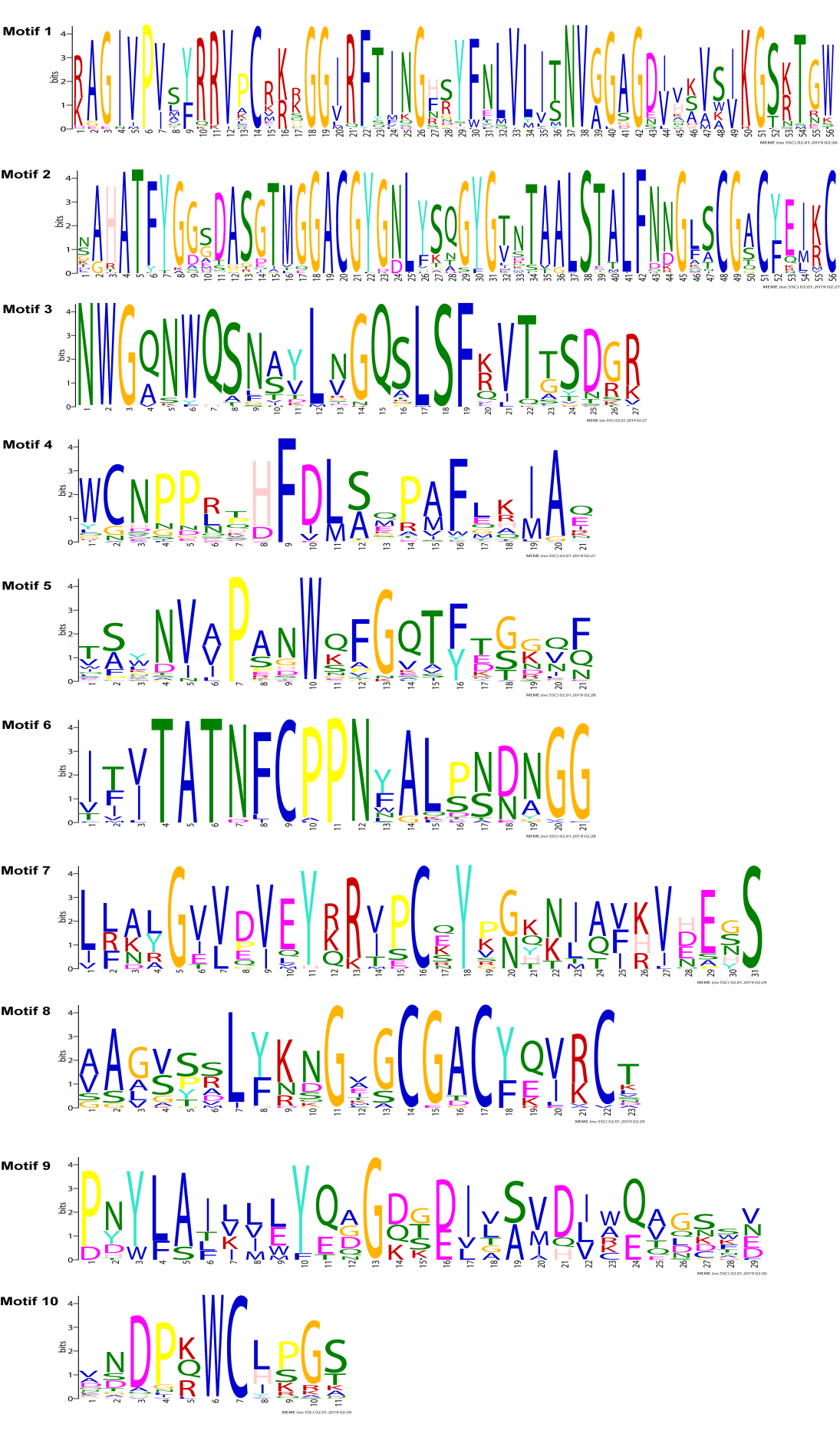


**Figure S3. Expression proﬁles of *GhEXP* genes in cotton ovules and fibres.** The heat map was constructed based on RNA-seq data in a publicly available database. Different colours represent the different expression levels of *GhEXP* genes. The 14 *expansin* genes verified by qRT-PCR are marked with black arrows.

**
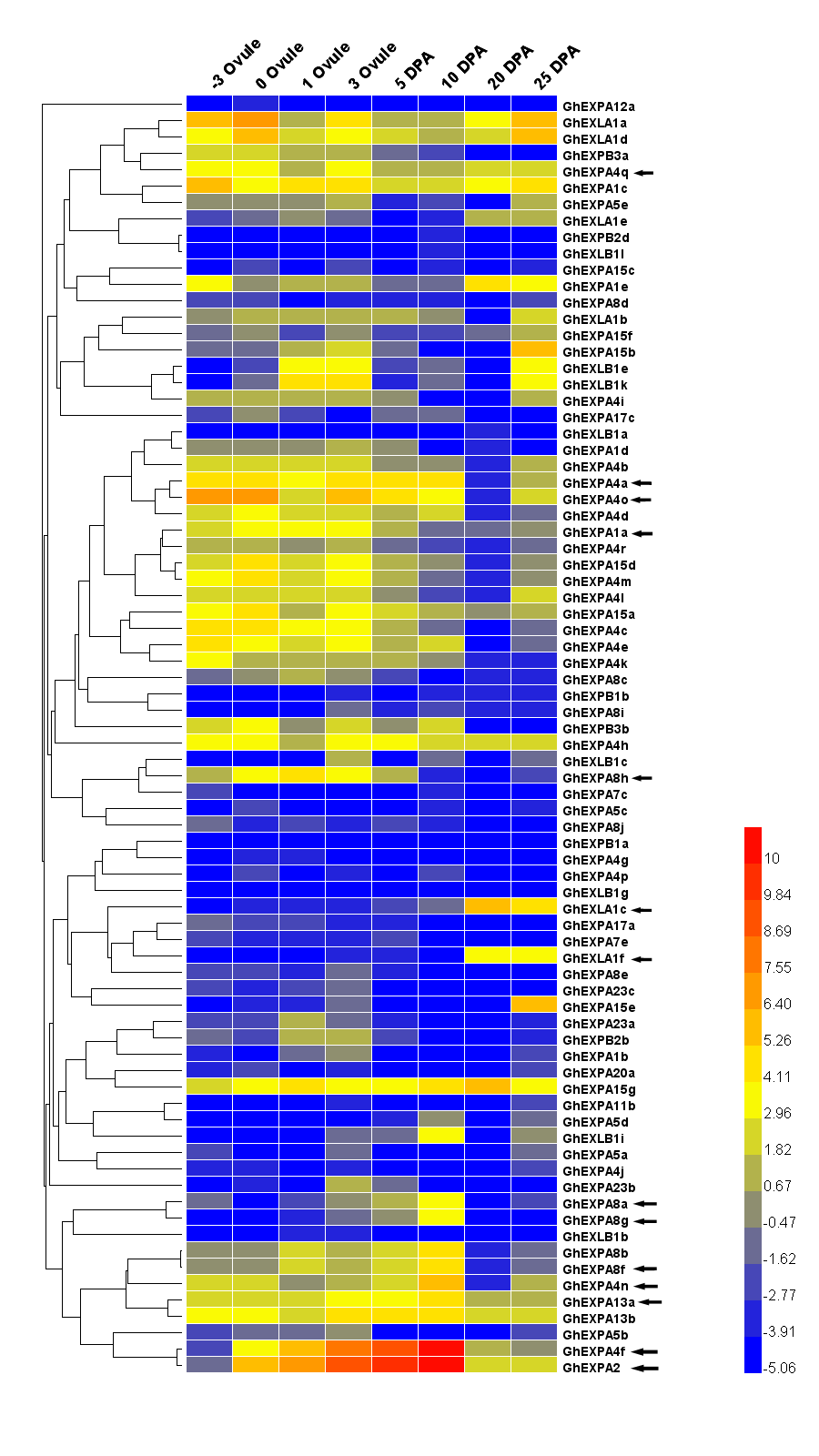
**

**Figure S4. Quantitative RT-PCR analysis of seven *GhEXP* genes at different stages of fibres and in different tissues.** The samples were collected at fibre stages of 0 DPA, 3 DPA, 5 DPA, 7 DPA, 10 DPA, 15 DPA, 20 DPA, and 30 DPA; the tissues included root, hypocotyl, stem, leaf, calycle, petal, pollen and stigma tissues. qRT-PCR experiments were performed with three independent replicates, and the error bars in this ﬁgure represent the SDs from three independent experiments.

**
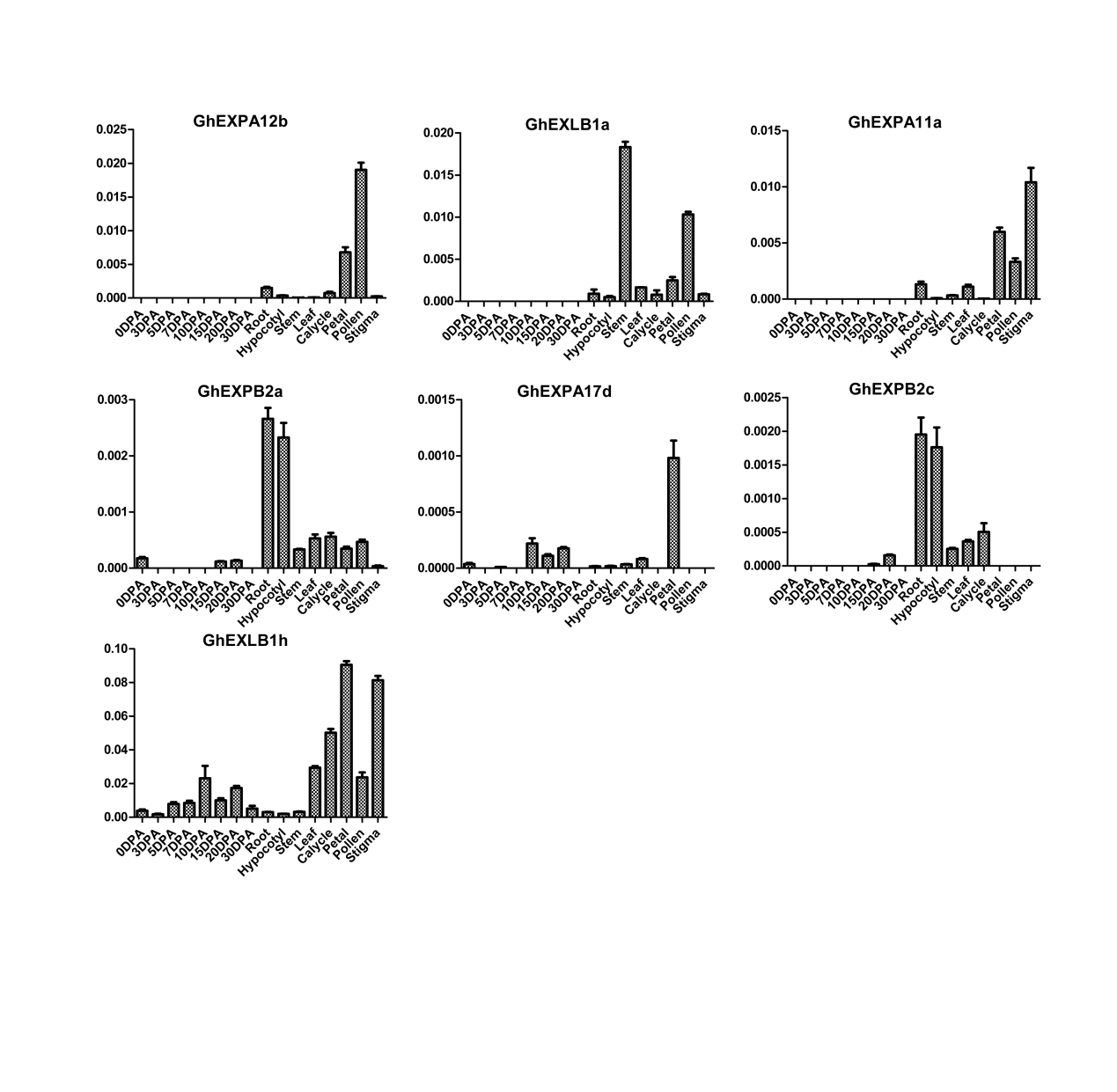
**

**Figure S5. qRT-PCR validation and transcriptome sequencing of 14 *GhEXPs* at different developmental stages of cotton fibres.** (a) qRT-PCR validation and transcriptome sequencing of three *GhEXP* genes highly expressed in the fibre initiation period. (b) qRT-PCR validation and transcriptome sequencing of nine *GhEXP* genes highly expressed at the fibre elongation stage. (c) qRT-PCR validation and transcriptome sequencing of two *GhEXP* genes highly expressed at the secondary wall synthesis stage. qRT-PCR experiments were performed with three independent replicates, and the error bars in this ﬁgure represent the SDs from three independent experiments. FPKM values represent the results of transcriptome sequencing.

**Figure S5.**


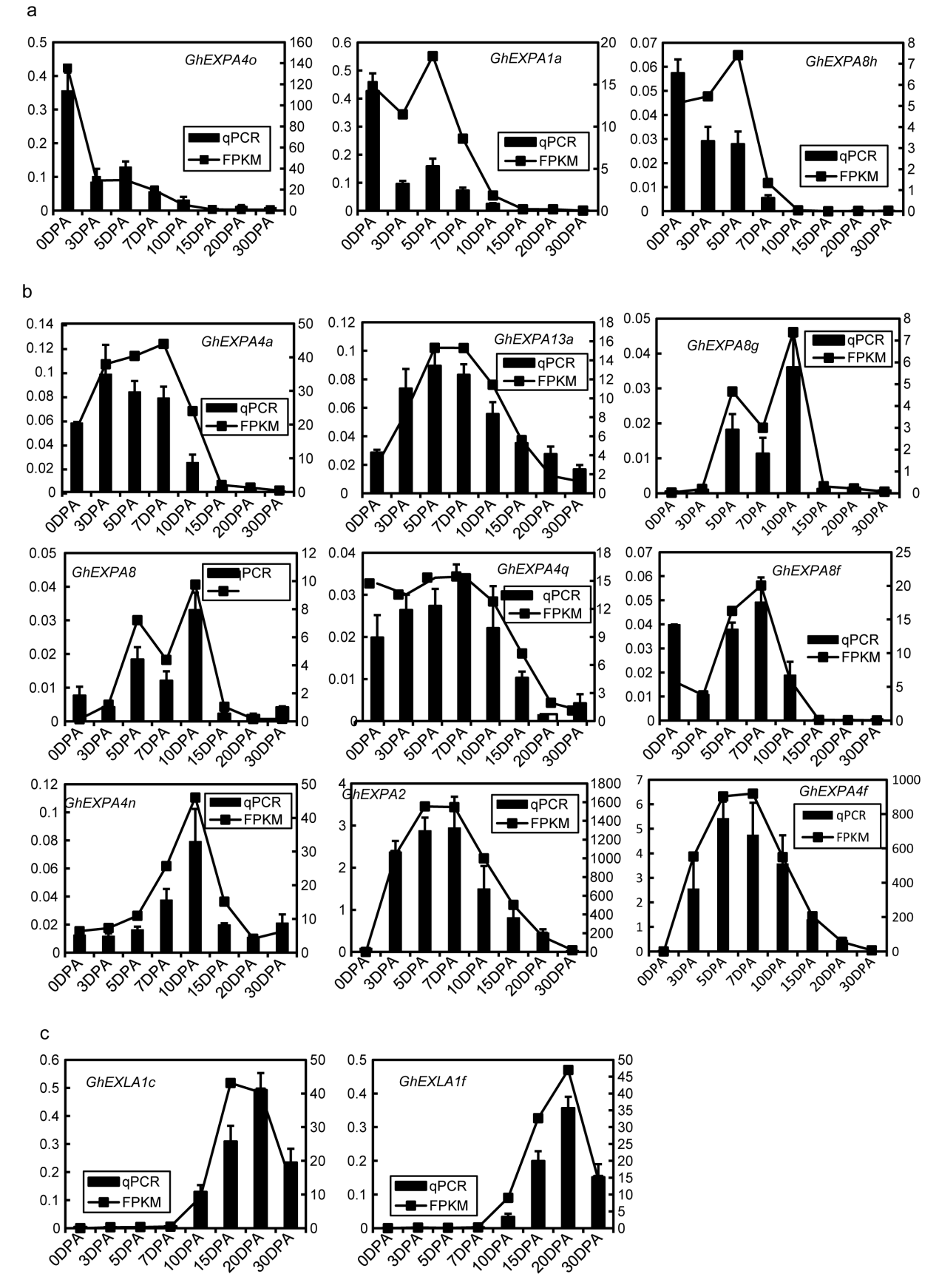


**Figure S6. Quantitative RT-PCR analysis of 14 cotton *GhEXP* genes in different tissues.** The different tissues included 11 samples: fibres at 0 DPA, 10 DPA, and 20 DPA as well as roots, hypocotyls, stems, leaves, calycles, petals, pollen and stigmas. qRT-PCR experiments were performed with three independent replicates, and the error bars in this ﬁgure represent the SDs from three independent experiments.


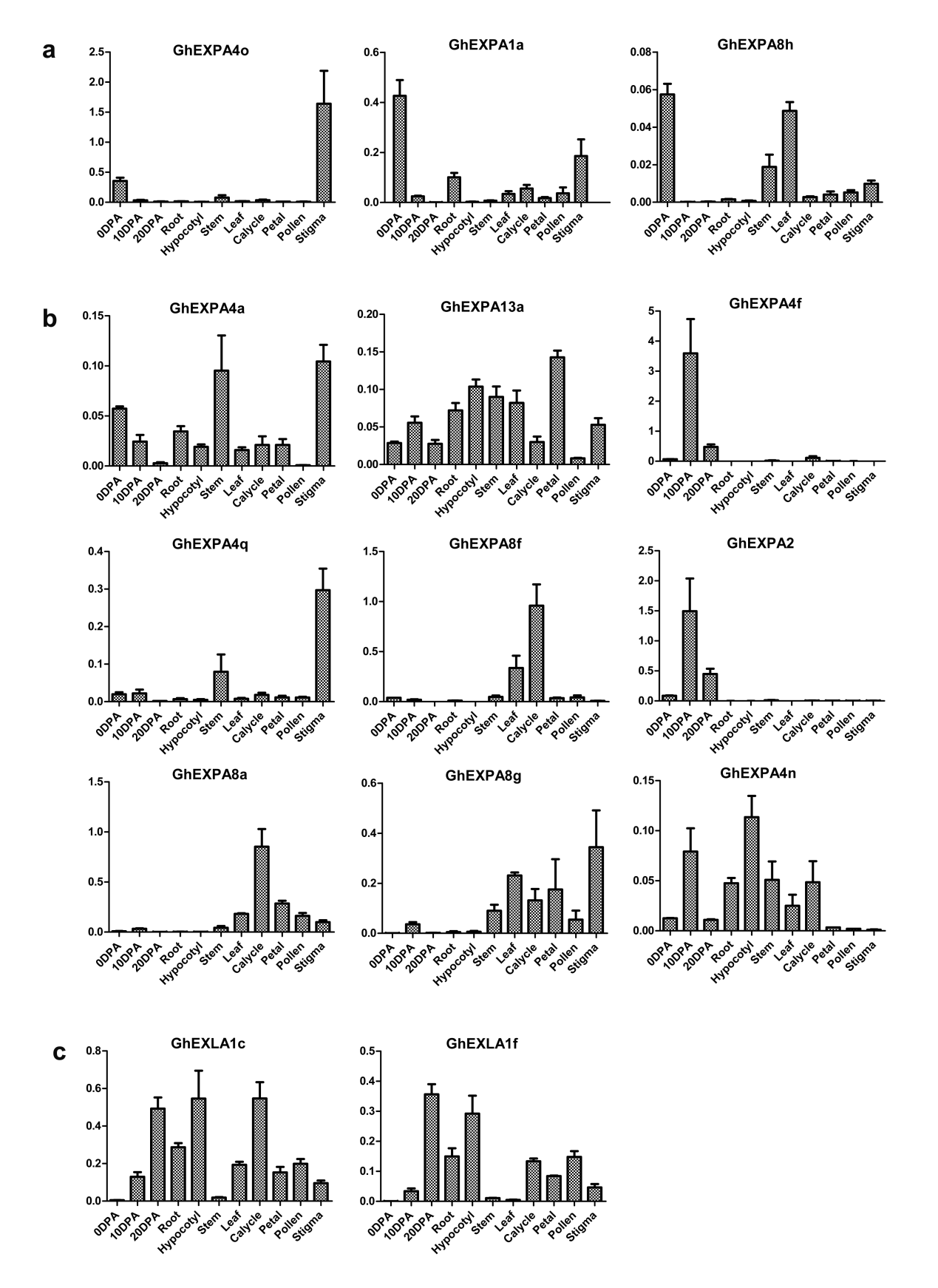

Supplement: Supplementary file 2 — Additional file 2: Fig. S1. Multiple sequence alignment of 93 GhEXP proteins. Fig. S2. Analysis of conserved motifs of GhEXP genes in cotton. Fig. S3. Expression profiles of GhEXP genes in cotton ovules and fibres. Fig. S4. Quantitative RT-PCR analysis of seven GhEXP genes in fibres at different stages and in different tissues. Fig. S5. qRT-PCR validation and transcriptome sequencing of 14 GhEXPs at different developmental stages. Fig. S6. Quantitative RT-PCR analysis of 14 cotton GhEXP genes in different tissues. [file 12870_2020_2362_MOESM2_ESM.docx]
